# Endogenous protein tagging coupled with a CRISPR screening approach identifies UBE3C as a potential MYC oncogene regulator

**DOI:** 10.1038/s41598-026-47974-w

**Published:** 2026-04-11

**Authors:** Marcel Seibert, Nina Kurrle, Sifora Kaleab, Frank Wempe, Ivana von Metzler, Hubert Serve, Frank Schnütgen

**Affiliations:** 1 Department of Medicine Hematology/Oncology, Goethe University Frankfurt, University Medicine Frankfurt, 60590 Frankfurt am Main, Germany; 2 German Cancer Consortium (DKTK), Partner Site Frankfurt/Mainz, and German Cancer Research Center (DKFZ), 69120 Heidelberg, Germany; 3 Frankfurt Cancer Institute, Goethe-University Frankfurt, 60596 Frankfurt am Main, Germany; 4 University Cancer Center Frankfurt (UCT), University Medicine Frankfurt, Goethe University, 60590 Frankfurt am Main, Germany

**Keywords:** Oncogene proteins, Haematological cancer, Cancer genomics

## Abstract

The transcription factor MYC is a key regulator of cellular proliferation and metabolism and is frequently dysregulated in malignancies such as multiple myeloma (MM). Despite its clinical relevance, direct therapeutic targeting of MYC remains limited, emphasizing the need to identify upstream regulators that control endogenous MYC expression. To systematically uncover such regulators, we developed a genome-wide CRISPR-Cas9 loss-of-function screening approach, employing a custom-engineered MM reporter cell line (RPMI8226-F11), in which oncogenic MYC protein was endogenously tagged with EGFP (referred to as GFP). This fluorescent readout enabled a direct, quantitative assessment of endogenous MYC expression levels. A pooled genome-wide sgRNA library was introduced, and cells were sorted based on GFP fluorescent intensity to reflect varying MYC levels. Next-generation sequencing of sgRNA distributions across sorted populations enabled the identification of candidate MYC regulators. Validation of screen hits, including the established MYC activator IRF4 and repressor FBXW7, confirmed the reliability of our system. To further dissect regulatory networks, we performed an overrepresentation analysis of target genes, which revealed the enrichment of Mediator complex subunits among MYC activators and ubiquitin–proteasome pathway components among MYC repressors. Functional validation of prioritized hits—MED30 (Mediator complex) and UBE3C (E3 ubiquitin ligase)—demonstrated a strong impact on endogenous MYC levels. Notably, the knockout of UBE3C markedly increased MYC expression, whereas its paralogs, UBE3A and UBE3B, showed no measurable effect, suggesting a specific regulatory role for UBE3C in MM cells. Together, our study provides a comprehensive CRISPR screen-based resource for the discovery of MYC regulators and highlights UBE3C as a potential therapeutic node for modulating MYC expression in MM.

**Supplementary Information:**

The online version contains supplementary material available at 10.1038/s41598-026-47974-w.

## Introduction

The transcription factor c-MYC (referred to as MYC) is a central driver of tumorigenesis in a wide range of malignancies, such as multiple myeloma (MM), orchestrating diverse cellular processes such as proliferation, metabolism, and apoptosis^1–3^. Due to its pivotal role in cancer biology, understanding the regulatory mechanisms governing MYC expression and activity is crucial. However, the intricate network of factors modulating MYC remains incompletely understood.

In MM but also other malignancies, targeting MYC as a therapeutic strategy has been explored with inhibitors such as bromodomain and extra-terminal motif (BET) protein inhibitors (BETis), like JQ1, showing promising preclinical efficacy^3–5^. However, BETis are not MYC-specific and affect broader transcriptional networks, limiting their clinical success. A more direct approach was pioneered with Omomyc, a dominant-negative bHLHLZ variant that dimerizes with MYC and blocks its binding to E-box DNA sequences by disrupting MYC–MAX interactions and promoting the formation of Omomyc homo- and heterodimers with MYC and MAX^6,7^. Omomyc has been developed into the clinical agent OMO-103 and was recently tested in a first-in-human phase I trial^8^ with a manageable safety profile and preliminary signs of target engagement. While promising, challenges remain for its application in MM, including efficient delivery and potential context-dependent efficacy, but its emergence underscores the growing feasibility of directly targeting MYC. Alternative strategies in MM have thus continued to target chromatin modifiers, that have been identified as potential targets for indirect MYC suppression, e.g. via the interferon regulatory factor 4 (IRF4)-MYC axis^9,10^. Another approach for MYC suppression focused on destabilizing MYC by promoting its ubiquitination and proteasomal degradation, as seen with the E3 ubiquitin ligase F-box/WD repeat-containing protein 7 (FBXW7)^11^. However, although several potential MYC-targeting strategies have been described, only a limited number of mono- and combination therapies have progressed to clinical trials, highlighting the urgent need for drug development for effective strategies targeting the MYC regulating network.

Functional genomic approaches, particularly CRISPR-based screening technologies, offer powerful tools to systematically identify genetic regulators of key oncogenic pathways. In this study, we therefore employed a genome-wide CRISPR loss-of-function screen to uncover regulators of native MYC expression. To investigate MYC regulation in its native genomic context, we developed an innovative strategy to first endogenously tag MYC with an N-terminal localization and purification (nLAP) tag^12^, containing an EGFP (referred to as GFP) marker in the MM cell line RPMI8226. Our endogenous approach ensures that native MYC expression levels directly correlate with GFP fluorescence allowing precise and physiologically relevant assessment of both MYC activation and repression.

In addition to known MYC regulators, such as IRF4 or FBXW7, that were identified by our screen and thereby provide evidence that our approach meets the desired reliability and reproducibility, we provide exploratory examples of potential MYC modulators that warrant further investigation on MYC regulatory networks. A particular strength of our approach lies in its ability to detect both MYC activators and repressors, enabling a comprehensive and physiologically relevant dissection of MYC regulation. These results not only offer insights into the methodological advancements necessary for endogenous and precise functional studies on MYC but also provide a broader understanding of MYC regulation in MM and pave the way for future studies in other MYC-driven cancer entities.

## Methods

### Cell culture

The human multiple myeloma (MM)-derived cell lines RPMI-8226, LP1, and OPM2 were obtained from the Deutsche Sammlung von Mikroorganismen und Zellkulturen (DSMZ, Braunschweig, Germany) and were cultured in RPMI-1640 medium (Gibco/Thermo Fisher Scientific, Darmstadt, Germany #21875034), supplemented with 10% (v/v) FCS (Sigma-Aldrich, USA), 100 U/mL penicillin and 100 μg/mL streptomycin (1 × P/S) (Thermo Fisher Scientific, USA #15140122). For lentivirus production, Lenti-X HEK293T cells (Takara Bio Europe SAS, Saint-Germain-en-Laye, France, #Z2180N) were cultured in high glucose DMEM 4.5 g/L D-glucose medium (DMEM 4.5 g/L D-glucose, Gibco/Thermo Fisher Scientific, #41965-039), supplemented with 10% (v/v) FCS (Sigma-Aldrich, USA), and 1 × P/S. All cell lines were cultured at 37 °C in a humidified 5% CO_2_ incubator. All multiple myeloma cell lines, that were used for CRISPR/Cas9-mediated gene knockouts, stably express spCas9 generated beforehand by lentiviral transduction using the plasmid pLentiCas9-Blast (Addgene, Watertown, MA, USA #52962).

### Cell modifications (CRISPR/spCas9-mediated gene knockout and endogenous tagging)

Individual gene knockouts were generated by lentiviral transduction of 0.25 * 10^6^ spCas9-expressing RPMI8226 cells in 1 mL standard RPMI1640 medium with pLentiCRISPRv2_ΔspCas9 encoding sgRNAs targeting targets genes (Table 1). The expression of sgRNAs was coupled to fluorescent BFP marker expression to enable BFP-gating to analyze only positively-transduced cell populations. We designed all sgRNAs using the Benchling software package (Benchling [Biology Software] (2023) retrieved from https://benchling.com). All oligonucleotides were obtained from Sigma-Aldrich and cloned in pLentiCRISPRv2_ΔspCas9, which is derived from the commercially available pLentiCRISPRv2 plasmid (Addgene #52961), in which the cDNA encoding spCas9 was removed and the cDNA encoding the puromycin resistance cassette was replaced by a BFP-encoding cDNA. The cloning of sgRNAs was performed according to the Golden Gate protocol^13^ into the BsmBI site. Vector carrying a non-target control (NTC) sequence was used as control (Table 1).

**Table 1:** Oligonucleotide (5’-3’ orientation). For sgRNA sequences, lowercase letters indicate the gene-specific sequences; uppercase letters indicate the overhangs required for cloning and transcription initiation.

sgRNA ID	5’-3’ sequences	Marker
NTC	Sense: CACCGttccgggctaacaagtcct Antisense: AAACaggacttgttagcccggaaC	BFP
MYC (sgRNA(1)) (*CRISPR KO sgRNA*)	Sense: CACCGgacgctgtgcccgcgggcg Antisense: AAACcgcccgcgggcacagcgtcC	RFP
MYC (sgRNA(t)) (*Tagging sgRNA*)	Sense: CACCGacgttgaggggcatcgtcg Antisense: AAACcgacgatgcccctcaacgtC	BFP
UBE3A (sgRNA(2))	Sense: CACCGatcctcatccctccaagaa Antisense: AAACttcttggagggatgaggatC	BFP
UBE3B (sgRNA(2))	Sense: CACCGcagagaaaactccggacat Antisense: AAACatgtccggagttttctctgC	BFP
UBE3C (sgRNA(2))	Sense: CACCGgacttcaagacgcggccca Antisense: AAACtgggccgcgtcttgaagtcC	BFP
MED30 (sgRNA(2))	Sense: CACCGcactgtctcctgcccgatg Antisense: AAACcatcgggcaggagacagtgC	BFP
IRF4 (sgRNA(2))	Sense: CACCGgccaagcagctcaccctgg Antisense: AAACccagggtgagctgcttggcC	BFP
FBXW7 (sgRNA(2))	Sense: CACCGgaacatggtacaagcccag Antisense: AAACctgggcttgtaccatgttcC	BFP
Human CRISPR knockout pooled library (Brunello)	Addgene #73,178	Puromycin

Cloned plasmids were verified by Sanger sequencing and Lenti-X HEK293T cells were used to produce lentiviral particles^14^ using the packaging plasmids pMD2.G (Addgene, #12259) and psPAX2 (Addgene, #12260). Successful transduction was verified by flow cytometry analysis for fluorescence (BD LSRFortessa Cell Analyzer (BD Biosciences, USA)). 24 h after transduction, media were replaced with fresh standard media, and cells were expanded until the days of the experiments. Knockout efficiency was confirmed by Sanger sequencing of PCR amplicons spanning the sgRNA target sites in genomic DNA from knockout (KO) and non-targeting control (NTC) cells. Chromatograms from KO samples displayed mixed base calls and a pronounced loss of signal quality at the expected cut site, consistent with indel formation by non-homologous end joining.

For endogenous MYC tagging, spCas9-expressing RPMI8226 cells were co-transfected with the target-(MYC)-specific sgRNA(t) containing plasmid (pLentiCRISPRv2_ΔspCas9_BFP) and a generic nLAP (EGFP, referred to as GFP) donor plasmid (pGTag Hygro EGFP nLAP + 1, Addgene, #194306), as described methodically in Thöne et al.^15^. Co-transfections were performed by electroporation using the Invitrogen Neon Transfection System according to manufacturer’s instructions for 100 µL NEON tips (5.6*10^6^ RPMI8226 cells/mL, 1200 V, 20 ms pulse width, 2 pulses, 10 µg DNA). The sgRNA sequences were designed to bind upstream of the initiating ATG start codon for N-terminal MYC tagging, initiating a DNA double-strand break at the target locus. The donor plasmid releases the tag, which is incorporated into the target locus via the NHEJ pathway, ensuring the original reading frame is preserved^15^. The donor plasmids were designed with a hygromycin selection marker. Selection for tagged MYC was done using 400 µg/mL hygromycin (Thermo Fisher Scientific, #10687010) and FACS (BD FACS Aria II (BD Biosciences, USA)) to isolate GFP-positive cells. For single clone sub-cultivation, hygromycin-selected MYC-tagged cells were diluted to a concentration of 30 cells/mL and plated in 96-well plates by limiting dilution. Individual clones were expanded by transferring them into higher volumes until sufficient cell numbers were available for analysis. To confirm the correct integration of the nLAP (GFP) tag, genomic DNA from individual clones was extracted and PCR amplification was performed on 5’- and 3’-junctions of the tag and gene using specific primers (Table 2). PCR was done to check for WT allele integrity. GFP fluorescence of tagged MYC was confirmed by flow cytometry (BD LSRFortessa Cell Analyzer (BD Biosciences, USA)). To validate MYC tagging functionally, tagged MYC-expressing RPMI8226 cells were treated with the known MYC suppressor JQ1 (500 nM, Sigma-Aldrich, USA, #SML0974) for 24–72 h and GFP fluorescence shifts were determined using flow cytometry (BD LSRFortessa Cell Analyzer (BD Biosciences, USA)). In addition, interaction of tagged MYC with the obligate heterodimerization partner (MAX)^16,17^ was tested by co-immunoprecipitation (CoIP) to validate the physiologically relevant functionality of our engineered fusion construct.

**Table 2:** Primer sequences (5’-3’ orientation).

Primer ID	5’-3’ sequences
MYC *forward*	5’-CAAGCCGCTGGTTCACTAAG-3’
MYC *reverse*	5’-GAAGGGAGAAGGGTGTGACC-3’
Generic tag *forward*	5’-CAGCTGCTGCTAAATTCGAG-3’
Generic tag *reverse*	5’-GACATATCCACGCCCTCCTA-3’

### Genome-wide CRISPR loss-of function screen and subsequent cell sorting (FACS)

To identify MYC driver genes as potential therapeutic targets, we utilized a genome-wide CRISPR loss-of-function screen in a RPMI8226 cell subclone (RPMI8226-F11 cells) expressing endogenously nLAP-tagged MYC (referred to as MYC-GFP). RPMI8226-F11 cells were transduced with the genome-wide Brunello sgRNA library (Addgene #73178), which contains 76,441 sgRNAs targeting 19,114 genes, along with 1000 non-targeting controls. To ensure single sgRNA integration per cell, transduction was performed at a low multiplicity of infection (MOI = 0.2), maintaining a 500-fold library coverage. Following transduction, cells were cultured for 4 days under puromycin selection (2 µg/mL) to enrich for sgRNA-positive cells. For MYC expression analysis, dead cells were removed via Ficoll gradient centrifugation, and viable cells were sorted into MYC-GFP-high, -intermediate, and -low expression fractions based on GFP fluorescence using a BD FACS Aria II flow cytometer. Based on considerations of signal dilution versus sampling noise, we used 5% high and 5% low gates (≈2.5 × 10^6^ cells each from 50 × 10^6^ total sorted cells), which ensured ~ 30-fold library coverage and high specificity. Sorted cell populations were immediately lysed for genomic DNA (gDNA) isolation, followed by next-generation sequencing (NGS) analysis. sgRNA sequences were barcode-labeled to enable quantification of their representation across sorted fractions, allowing for the identification of candidate MYC-regulating genes. To enhance data robustness, all experiments were conducted in triplicates. Raw sequencing data have been deposited in the NCBI database under BioProject accession PRJNA1354411. Processed datasets are available in The BioGRID ORCS repository (Seibert2025).

### NGS sequencing and data analyses

To identify enriched or depleted sgRNAs in different MYC expression fractions, sgRNA-encoding regions from sorted cell populations (MYC-GFP-high, -low, and -intermediate) were barcode-labeled and subjected to next-generation sequencing (NGS) using the Illumina NextSeq 500 platform. PCR amplification of sgRNA-encoding regions was performed using the Q5 High-Fidelity 2X Master Mix (New England Biolabs, Germany, #M0492L). Up to 80 parallel PCRs were performed per sample to ensure sufficient amplification, using staggered P5 primers (1–9 bp random sequences) and nine distinct P7 primers for barcode incorporation. Amplified products were pooled, quantified densitometrically, and set to 60 ng/µL before purification with AMPure-XP beads (Beckman Coulter).

NGS was performed on an Illumina NextSeq 500 platform using a dual-indexed, paired-end sequencing approach. Libraries were prepared according to the Illumina NextSeq 500/550 high-output kit protocol, with 150-cycle read 1, 8-cycle index reads (i7 and i5), followed by 150-cycle read 2. A 30% PhiX control library was added to ensure balanced fluorescence signals. Bioinformatic analysis was performed by enGene Statistics GmbH following established quality control metrics. Mapping rates exceeding 65% were required to ensure sequencing reliability, while sgRNA representation was assessed using the Gini index (expected ranges: ~ 0.1 for intermediate samples, 0.2–0.3 for high-/low-sorted samples). Additionally, the fraction of undetected sgRNAs was kept below 1%. Mapping rates across all samples exceeded 60%, confirming the successful preparation and sequencing of samples (Fig. S2B). Except for the GFP-high sample from replicate 1, all samples exhibited a Gini index below 0.13, indicating a relatively uniform distribution of sequencing reads. As expected, the GFP-low and GFP-high sorted fractions showed slightly increased Gini indices compared to the intermediate control samples, reflecting enrichment effects due to sorting. While this trend was evident in replicate 1, it was less pronounced in the second and third replicates, although all indices remained within the defined quality thresholds (Fig. S2C). Additionally, apart from the GFP-high sample of replicate 1, all samples contained fewer than 1% missing gRNAs (defined as gRNAs with zero counts; Fig. S2D). Statistically significant MYC regulators were identified by comparing sgRNA abundance across sorted fractions, with β-score calculations indicating enrichment or depletion in specific MYC expression groups.

For pathway analysis, genes identified in the CRISPR screen were ranked based on their β-scores. Genes with the highest β-scores (for sgRNA enrichment) or the lowest β-scores (for sgRNA depletion) that clearly separated from the bulk genes were analyzed using overrepresentation analysis (ORA). ORA was conducted using the web-based tool g:Profiler (version e106_eg53_p16_65fcd97)^18^ to calculate the enrichment *p* value based on the likelihood of identifying an enrichment of a group of genes that belong to a specific functional group within a dataset, as described by Boyle et al.^19^. This allows for pathway enrichment analysis by integrating multiple gene annotation databases, including the Comprehensive Resource of Mammalian Protein Complexes (CORUM) and Gene Ontology (GO): Biological Processes (BP) and Cellular Components (CC), to identify functional categories associated with MYC activation or repression. For enrichment *p* value calculation, the Benjamini–Hochberg FDR multiple testing correction method with a significance threshold of 0.05 was applied. The extent, to which a specific functional category is represented in the dataset, was quantified using the gene ratio, calculated as the number of identified genes within a specific pathway divided by the total number of genes known to belong to that pathway. A gene ratio of 1 indicates that all genes in a given pathway were identified in the screen, suggesting high specificity and significance, which is further supported by the corresponding enrichment *p* value.

### Cell lysis, CoIP, SDS-PAGE, and Western blot

For protein lysates, 1 * 10^6^ cells were lysed by adding 50 µL of SDS lysis buffer (100 mM Tris HCl pH8 (Carl Roth GmbH, #9090.3), 150 mM NaCl (Riedel-de-Haёn, #31434), 10 mM EDTA pH8 (PanReac AppliChem, #A4892,0500), 10% SDS (MP Biomedicals #04811033-CF), freshly supplemented with protease inhibitors (cOmplete, Mini, EDTA-free Protease Inhibitor Cocktail, Roche/Merck, #11836170001). SDS lysates were cleared by centrifugation. Protein concentrations were determined by Lowry assay (DC Protein Assay Reagent A and B, Biorad, USA) according to the manufacturer’s instructions. Equal amounts of proteins were prepared for SDS-PAGE and Western blot by boiling in 4 × SDS loading dye (250 mM Tris HCl pH 6.8 (Carl Roth GmbH, #9090.3), 8% SDS (MP Biomedicals #04,811,033-CF), 40% Glycerol (Carl Roth GmbH, #3783.1), 0.2% Bromphenol blue (Sigma-Aldrich, #B0126)). For CoIP of GFP-tagged MYC and its interaction partner MAX, 30 * 10^6^ cells were collected from cell culture, washed with cold 1 × PBS, and lysed in CoIP buffer (10 mM Tris–HCl pH8 (Carl Roth GmbH, #9090.3), 150 mM NaCl (Riedel-de-Haёn, #31,434), 5 mM EDTA pH8 (Solution, PanReac AppliChem, #A4892,0500), 0.5% Triton X-100 (Fluka, #93420), 60 mM Octyl- β-D-glucopyranoside (Sigma-Aldrich, #O8001) supplemented with protease inhibitors (cOmplete, Mini, EDTA-free Protease Inhibitor Cocktail, Roche/Merck, #11836170001). After a 30-min incubation on ice, the lysates were centrifuged, and protein concentrations were determined using the Bradford assay. A total of 4000 μg of protein was incubated with 25 μL GFP-Trap magnetic agarose beads (ChromoTek, #gtma), or 25 µL binding control beads “IgG” (ChromoTek, #bmab) overnight at 4 °C with gentle rotation. The beads were then washed five times with CoIP buffer using a magnet. Finally, proteins were eluted by boiling in 4 × SDS loading dye. Equal protein amounts of the lysates were analyzed by SDS-PAGE and Western blot.

### Antibodies

For Western blot detection, the anti-MYC and anti-MAX antibodies were diluted 1:1000 in TBST (TBS buffer B (Zytomed Systems, #ZUC066), containing 0.05% (v/v) Tween-20 (Carl Roth GmbH, #9127.1) and 0.5% (v/v) NaN_3_ (Sigma, #S2002)); anti-GAPDH and anti-Vinculin antibodies were applied in a 1:20,000 dilution. GAPDH (Abcam (6C5), Mouse #ab8245), MYC (Cell Signaling (D84C12) Rabbit mAb #5605), MAX (Proteintech, Rabbit pAb, #10426-1-AP), Vinculin (Abcam (SPM227) Mouse mAb #ab18058), FBXW7 (Proteintech, Rabbit pAb, #28424-1-AP), IRF4 (Proteintech, Mouse mAb, #66451-1-Ig), MED30 (Proteintech, Rabbit pAb, #16787-1-AP), UBE3C (Proteintech, Rabbit pAb, #12333-1-AP).

## Results

The most common event that triggers neoplastic transformation in malignancies like MM is the deregulation of oncogenic MYC, bringing MYC into focus as a therapeutic target protein. To investigate MYC regulation in its native genomic context, we endogenously tagged MYC with GFP in the MM-derived cell line RPMI8226, allowing direct fluorescence-based monitoring of MYC expression. In the first step, we validated the correct integration and functionality of the tag. Subsequently, single-cell clones were generated and characterized to ensure precise MYC-GFP expression. A well-characterized clone was then selected for a genome-wide CRISPR knockout screen to identify regulators of MYC expression.

### Generation and characterization of single-cell clones expressing MYC-GFP

**Fig. 1:**
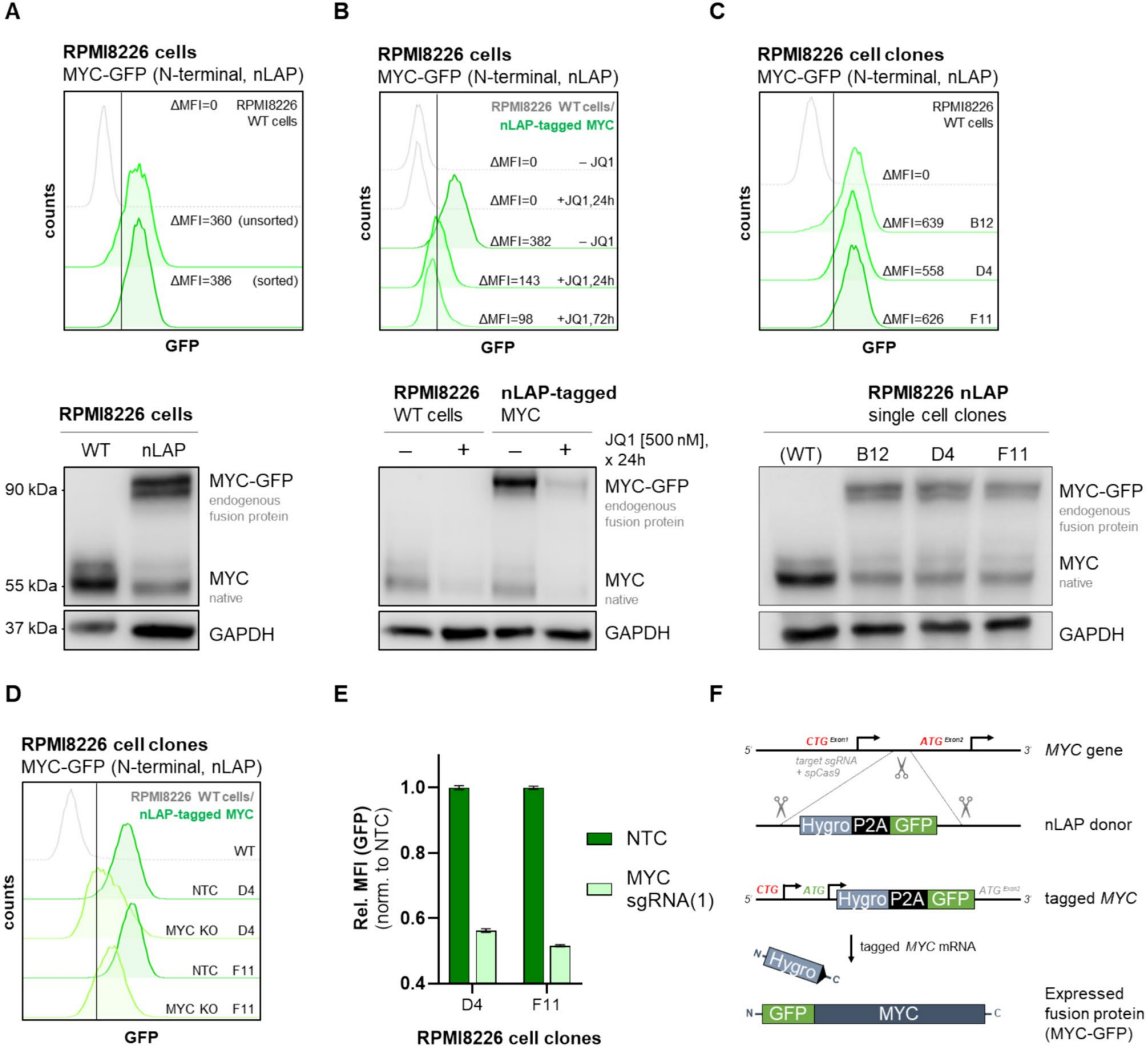
Validation of endogenously nLAP-tagged MYC expression and characterization of MYC-GFP expression in RPMI8226 subclones. (**A**) Representative flow cytometry analysis of GFP signals in RPMI8226 cells expressing nLAP-tagged MYC. Differences in GFP mean fluorescence intensity (ΔMFI) values were determined, with wild-type (WT) cells serving as GFP-negative controls. Western blot analysis confirming the expression of nLAP-tagged MYC. The upper band (~ 90 kDa) corresponds to MYC-GFP, while the lower band (~ 55 kDa) represents native MYC. (**B**) MYC expression analysis upon transcriptional MYC repression using 500 nM JQ1. Flow cytometry analysis shows a reduction in GFP fluorescence after 24 h and 72 h of JQ1 treatment. Western blot analysis confirms MYC downregulation in JQ1-treated cells. (**C**) Representative flow cytometry analysis of GFP signals in MYC-GFP-expressing RPMI8226 subclones B12, D4, and F11 (upper panel). GFP-positive signals were detected, and ΔMFI values were calculated by subtracting the GFP MFI of GFP-negative WT (control) cells. Western blot analysis of RPMI8226 subclones confirmed MYC-GFP expression (lower panel) (**D**) Representative flow cytometry analysis of GFP signals in RFP-positive MYC knockout (KO) RPMI8226 cell clones, RFP-gated to exclude non-transduced cells in GFP channels. WT cells served as GFP/RFP-negative controls. (**E**) Quantification of GFP MFI values in MYC KO RPMI8226 subclones compared to their respective non-target control (NTC) subclones. (**F**) Schematic representation of endogenous MYC tagging. The nLAP donor DNA fragment was inserted in-frame 5’ upstream of the canonical ATG start codon in exon 2 (shown in red) following sgRNA/CRISPR-mediated double-strand break induction and repair. Translation of the tagged MYC mRNA, initiated either from the nLAP ATG (green) or from the non-canonical CTG start codon in exon 1, results in the production of the MYC–GFP fusion protein. The P2A self-cleaving peptide subsequently releases the hygromycin-resistance protein, thereby preserving the native transcriptional regulation of MYC. ΔMFI values were calculated as the difference between the MFI values of MYC KO samples and their corresponding NTC samples. Shown are mean ± SEM. Western blots were probed with an anti-MYC antibody and with anti-GAPDH serving as a loading control. Data were analyzed using FlowJo v7.6.5 software.

To confirm successful endogenous tagging of MYC, flow cytometry analysis revealed detectable GFP fluorescence in RPMI8226 cells co-transfected with MYC-targeting sgRNA and the generic donor plasmid (ΔMFI = 360, compared to WT RPMI8226 (control) cells), indicating expression of the endogenous MYC-GFP fusion protein (Fig. 1A). The highest 5% of GFP-positive cells were additionally sorted to isolate successfully MYC-GFP expressing cells, resulting in a slight increase of GFP fluorescence (ΔMFI = 386). Western blot analysis further confirmed the presence of both native MYC and endogenously tagged MYC–GFP at approximately 55 and 90 kDa, respectively (Fig. 1A, lower panel). The simultaneous detection of MYC–GFP and native MYC proteins indicates partial, likely monoallelic, integration of the tagging cassette, which is consistent with the known polyploid and translocated MYC locus configuration in RPMI8226 cells, and with the low efficiency of homozygous tagging previously reported for this strategy^15^. In the pooled population prior to subcloning, MYC–GFP appeared more strongly expressed than the native protein (Fig. 1A, B), whereas this difference was no longer observed in single-cell–derived clones (Fig. 1C). Such variation likely reflects differences in allele activity within the heterogeneous pool, possibly related to copy number or chromosomal context, and does not affect the functional integrity of the tagged allele used for subsequent experiments. To ensure that GFP fluorescence accurately reflected MYC expression, the BET inhibitor JQ1, known to suppress *MYC* transcription^4,20^, was applied for 24 h and 72 h. Flow cytometry and Western blot analysis demonstrated a corresponding decrease in GFP fluorescence and MYC expression upon JQ1 treatment, confirming the functional correlation between GFP fluorescence intensity and endogenous MYC expression levels (Fig. 1B). Following initial validation, single-cell clones were derived from the GFP-positive cell pool to ensure genetic uniformity and consistent expression of MYC-GFP. PCR analysis confirmed the successful integration of the nLAP-tag at the endogenous MYC locus in three selected clones (B12, D4, and F11) (Supplementary Table S1). Flow cytometry and Western blot analysis of these three individual subclones demonstrated consistent GFP fluorescence and MYC-GFP expression, respectively (Fig. 1C). To further validate the correct tag integration and correlation of MYC expression and GFP fluorescence, a CRISPR-sgRNA-mediated MYC knockout was performed. An RFP marker was coupled to MYC-targeting sgRNA expression to enable the gating of RFP-positive cells when analyzing shifts in GFP fluorescence upon MYC knockout by flow cytometry. Targeting MYC directly leads to a marked reduction in GFP fluorescence compared to non-target control (NTC) cells, suggesting that knockout of MYC directly affects GFP fluorescence intensity in the tested subclones D4 and F11 (Fig. 1D and E). Additionally, to verify that the EGFP tag does not perturb MYC function, we performed co-immunoprecipitation (CoIP) of MYC (MYC-GFP) with its known interaction partner MAX in subclone F11. The successful co-precipitation confirmed that the bulky EGFP moiety does not interfere with MYC–MAX heterodimer formation (Supplementary Fig. S1). Figure 1F schematically illustrates the endogenous MYC tagging strategy. The nLAP donor DNA fragment was inserted in-frame 5′ upstream of the canonical ATG start codon in exon 2 of the MYC gene following sgRNA/CRISPR-mediated double-strand break induction and repair, thereby placing expression of the nLAP–MYC fusion under the control of the endogenous MYC promoter. Translation of the tagged MYC mRNA, initiated either from the donor-derived ATG or from the non-canonical CTG start codon in exon 1, results in MYC–GFP expression. The P2A peptide sequence mediates co-translational cleavage, releasing the hygromycin-resistance protein and preserving native MYC transcriptional regulation (see Fig. 1F and Table S1).

**Fig. 2:**
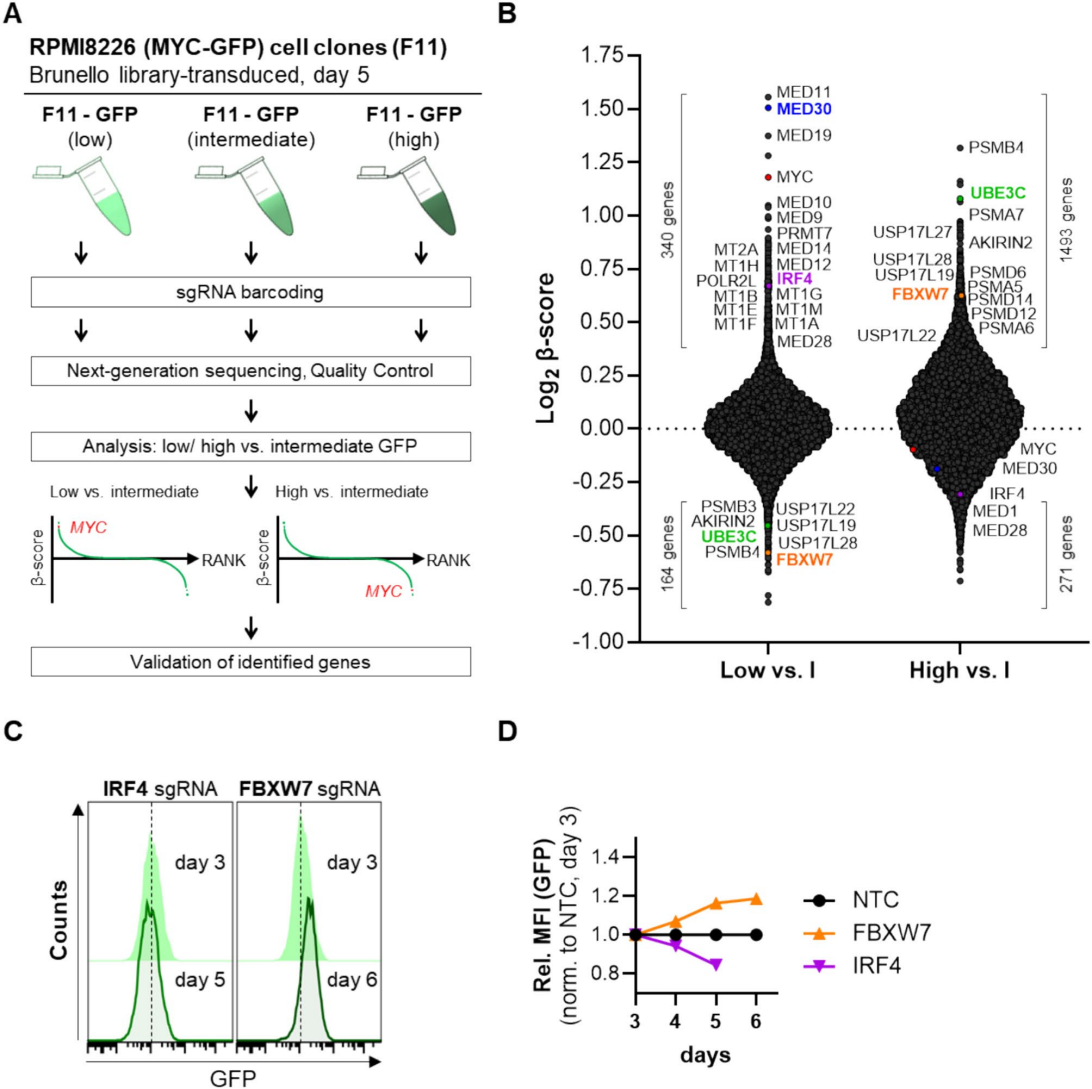
Screening for MYC-regulating driver genes. (**A**) Schematic overview of the MYC screen. F11 cells transduced with the Brunello library were sorted into three fractions: 5% GFP-low, 5% GFP-high, and 90% intermediate (control) GFP-positive cells. Barcoding of sgRNA sequences in each fraction allowed for the identification of genes potentially involved in MYC regulation by comparing the control fraction with the GFP-low or GFP-high fractions. (**B**) Genes identified in the screen are ranked according to their β-scores, representing the log-transformed ratios of GFP-low or GFP-high fractions relative to the intermediate control. Positive β-scores indicate genes whose knockout resulted in positive sgRNA selection (increased MYC expression), while negative β-scores indicate genes showing the opposite (decreased MYC expression after knockout). As expected, sgRNAs targeting MYC (highlighted in red) were enriched in the GFP-low fraction and depleted in the GFP-high fraction. Candidate genes selected for validation are marked in distinct colors. (**C**) Representative flow cytometry analysis of GFP signals in BFP-positive-gated cells following candidate/internal control (IRF4/FBXW7) gene knockout. Fluorescence intensity was assessed on day 3 and day 5/day 6 post-transduction with the respective sgRNA. (**D**) Relative GFP MFI values upon knockout of selected candidate genes. Data were analyzed using FlowJo v7.6.5 software.

### Genome-wide CRISPR knockout screen in the MYC-GFP reporter clone F11

**Fig. 3:**
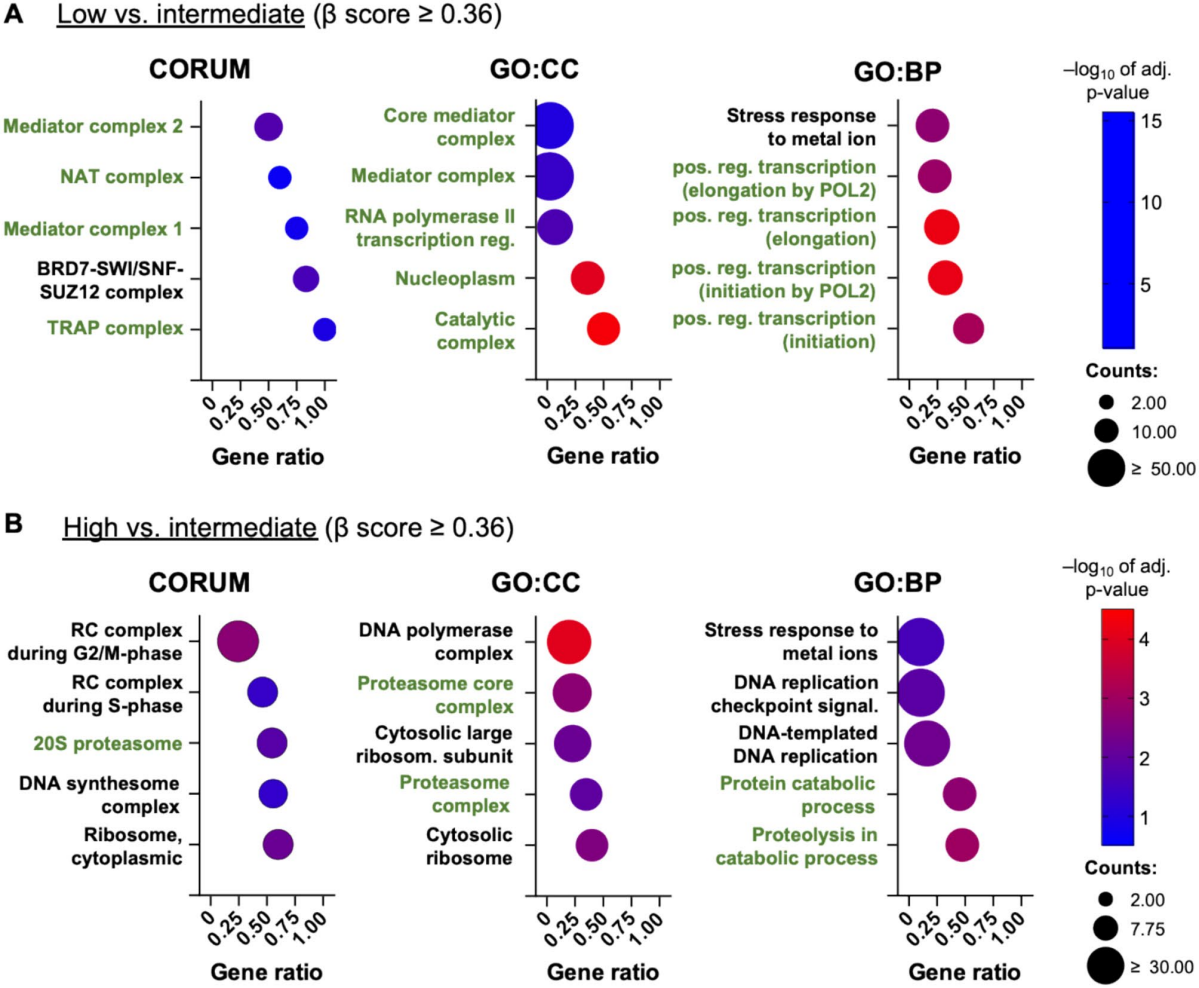
Functional enrichment analysis of MYC-regulating genes. Dot plots illustrate functional groups of (**A**) identified MYC-activator genes and (**B**) identified MYC-repressor genes, categorized based on shared associations in processes or complexes using data from the CORUM database or Gene Ontology (GO). Each dot represents a specific protein complex that contains the gene products, a Cellular Component (CC), or a Biological Process (BP), with the gene ratio indicating the proportion of identified genes relative to the total number of genes in the respective group. The dot size corresponds to the number of identified genes (counts), while the color reflects the calculated adjusted enrichment *p* value in negative log10 values. Although MYC-regulating genes could be assigned to different biological functions, MYC activators predominantly belong to the Mediator complex (**A**), whereas MYC-repressing genes mainly correspond to subunits of the proteasome complex (**B**). The respective complexes are highlighted in green in each analysis.

Based on the validation results, the F11 clone was selected for the genome-wide CRISPR screen. To systematically investigate MYC regulation, F11 cells were transduced with the genome-wide Brunello sgRNA library, leading to gene knockouts that could either enhance or suppress MYC expression, thereby altering GFP fluorescence intensity. Brunello sgRNA library-transduced F11 cells were sorted into MYC-high, -intermediate, and -low populations based on GFP fluorescence intensity. Flow cytometry plots confirmed successful sorting of distinct fluorescence populations (Figs. 3A and S2A). To assess the reliability of the screening data, a quality control analysis was performed to ensure sufficient sgRNA representation, minimal dropout rates, and high reproducibility (Fig. S2B–D). Predefined quality criteria were applied based on established computational analyses of CRISPR screens^21^.

Potential MYC regulators were identified based on over- or underrepresented sgRNAs in specific sorted cell fractions. This was quantified using the β-score, which reflects the relative enrichment or depletion of sequencing reads across conditions, alongside the *p* values (expressed as negative log_10_ (Wald *p* value)) that indicate the statistical significance of these changes. Genes identified as MYC activators were characterized by sgRNAs enriched in the GFP-low fraction relative to the intermediate control, resulting in positive β-scores. Conversely, MYC repressors were identified by the depletion of corresponding sgRNAs in the GFP-low fraction, yielding negative β-scores. The comparison of GFP-high with intermediate control fractions produced the inverse pattern, providing insights into MYC regulatory networks (Fig. 3). Analysis of the screen results identified several genes influencing MYC expression, visualized in a summarizing dot plot displaying these β-scores (Fig. 3B) and in volcano plots of the respective comparisons showing significantly enriched genes in the GFP-low and -high fraction (positive β-scores ≥ 0.585 (1.5 × f.c.), with log_10_ Wald *p* value ≥ 1.3 (0.05), Fig. S3). Among the identified hits, known regulators of MYC, such as IRF4 or FBXW7, were enriched or depleted in the respective fractions, serving as internal positive controls.

For internal experimental validation, IRF4 was selected as a representative MYC activator, while FBXW7 was chosen as a candidate MYC repressor. The functional relevance of these genes was tested using individual CRISPR/spCas9-mediated knockout assays in the RPMI8226-F11 MYC-GFP reporter cell clone (Figs. 3C, D and S4). The expression of sgRNAs targeting these MYC regulators was coupled to BFP expression, enabling the gating of successfully transduced cells. As expected, GFP fluorescence progressively decreased following knockout of IRF4, reaching 84.3% (normalized to NTC, day 5). In contrast, the knockout of FBXW7 led to an increase in GFP fluorescence to 118.7% (normalized to NTC, day 6) (Fig. 3C, D). These findings confirm that MYC expression responds in the expected direction upon the knockout of MYC regulators.

### Identification and functional analysis of MYC regulator genes

Beyond known MYC regulators, the screen identified 340 genes enriched in the low fraction and 1493 genes enriched in the high fraction whose positive beta scores were distinctly separated from the majority of bulk beta scores (cut-off value ≥ 0.36), revealing previously uncharacterized gene sets with a potential role in MYC regulation (Fig. 3B). To further expand the identification of MYC regulators, these genes were used for functional gene overrepresentation analysis (ORA). This analysis aimed to determine whether identified genes can be grouped and significantly associated with specific biological functions, according to the CORUM, GO:CC, and GO:BP databases (Fig. 3).

**Fig. 4:**
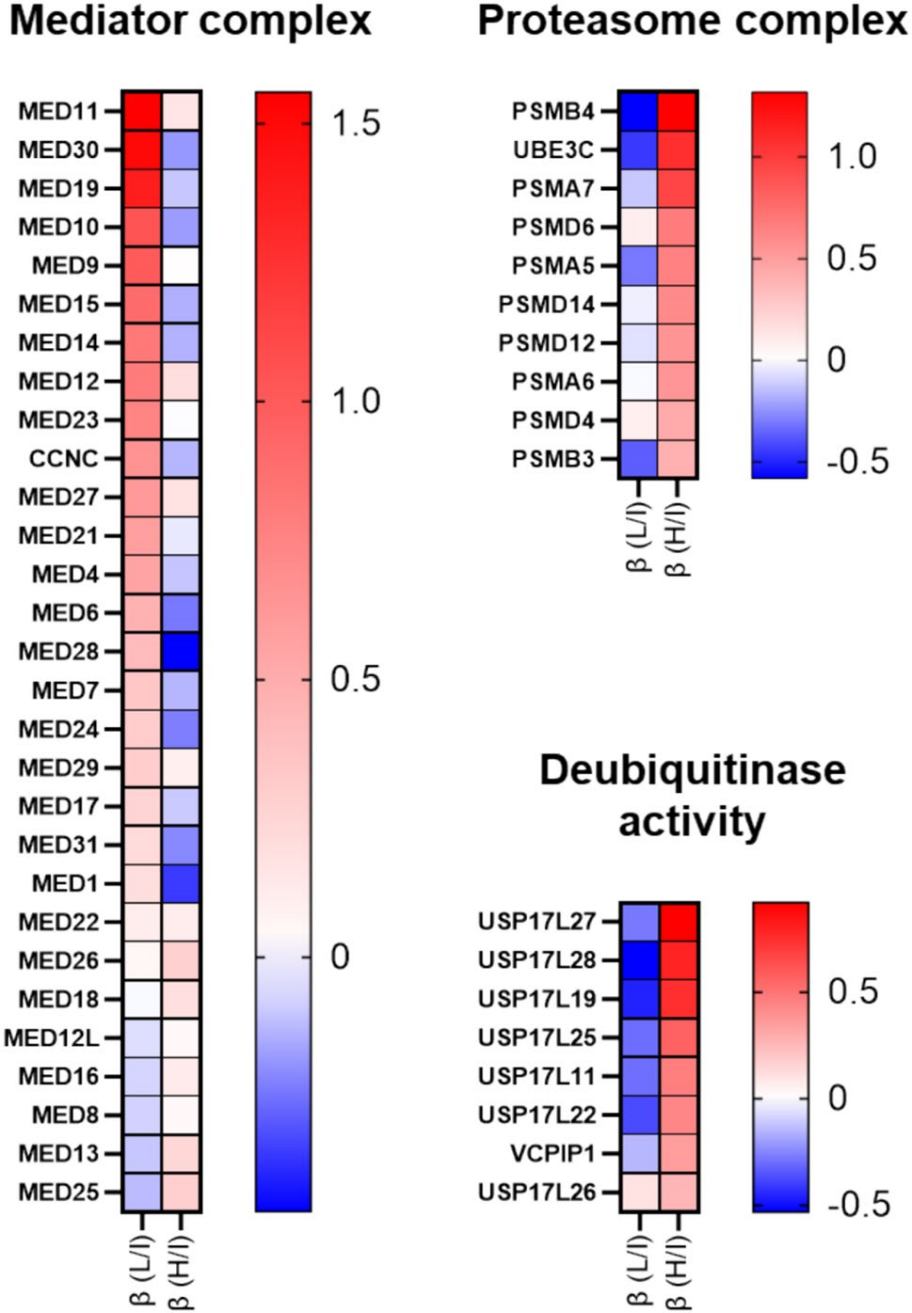
Log_2_ β-scores showing sgRNA enrichment/depletion in GFP-low vs. intermediate (L/I) and GFP-high vs. intermediate (H/I) analysis. Shown is the enrichment of sgRNAs targeting the Mediator complex subunits (MED) in the GFP-low fraction, sorted by their respective β-scores. In contrast, sgRNAs targeting proteasome subunits and genes encoding for proteins with deubiquitinase activity were depleted in L/I and enriched in H/L comparisons.

ORA revealed that genes encoding components of the Mediator (MED) complex were among the most significantly enriched activator genes, highlighting the potential role of this complex involvement in MYC activation (Fig. 3A). In contrast, the enrichment analysis of the high vs. intermediate fraction (potential MYC repressors) identifies clusters of genes encoding subunits of the proteasome (PSMA4/-6/-7, or PSMB4), UBE3C and AKIRIN2 reinforcing the role of proteasomal degradation in MYC regulation (Fig. 3B). This result was further supported by a cluster of genes belonging to proteolytic/catabolic processes containing ubiquitin specific peptidase 17 like (USP17Lx) family members associated with deubiquitinase activity. Altogether, these results suggest that MYC repression is primarily mediated through a ubiquitin-dependent degradation pathway. Although several protein clusters were identified by the ORA (Fig. 3), most hits mapped to two main functional groups—the Mediator (MED) complex and the proteasome—highlighting their role in MYC regulation. To prioritize key regulators, we compared beta-scores from low (L) vs. intermediate (I) and high (H) vs. intermediate (I) analyses. MYC activators showed positive beta-scores in L vs. I and negative in H vs. I, while MYC repressors showed the opposite. We focused on genes with the largest bidirectional separation, identifying MED30 and UBE3C as potential MYC activator and repressor, respectively (Fig. 4, highlighted in Fig. 2B).

**Fig. 5:**
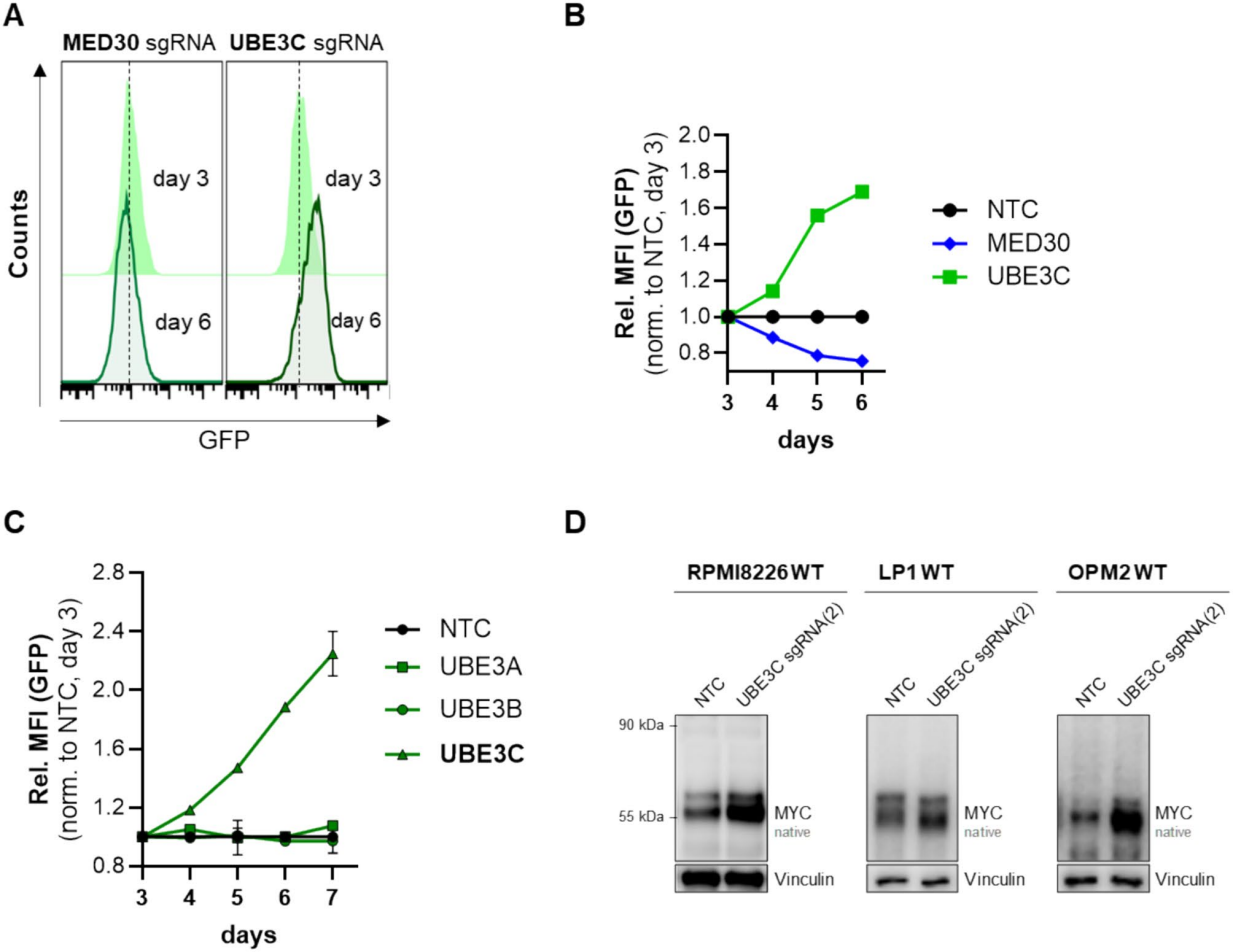
Potential role of MED30 and UBE3 paralogs as regulators of MYC. (**A**) Representative flow cytometry analysis of GFP signals in BFP-positive-gated MED30- and UBE3C-knockout cells on day 3 and 6 after transduction with the respective sgRNA. (**B**) Relative GFP MFI values upon knockout of selected candidate genes. (**C**) Comparison of relative GFP MFI values upon knockout of UBE3 paralogs (UBE3A, -B, -C). Shown are mean ± SD of two individual experiments. Data were analyzed using FlowJo v7.6.5 software. (**D**) Western blot analysis of native MYC expression after transduction with UBE3C-targeting sgRNAs in three different multiple myeloma cell lines. Blots were probed with an anti-MYC antibody; anti-Vinculin served as a loading control.

To validate the gene cluster analysis, representative candidates from the identified cluster were selected for further investigation, focusing on MED30 from the Mediator complex and UBE3C as a component of a ubiquitin-/proteasome-mediated degradation pathway. Remarkably, UBE3C is not a permanent part of the proteasome, but a non-stoichiometric associated protein that dynamically binds to it^22,23^. Furthermore, a recent study already demonstrated that the UBE3C paralog UBE3B regulates MYC in lymphoma by mediating its degradation^24^, suggesting a comparison of the three paralog UBE3A, -B, and -C in MYC regulation in myeloma cells. Therefore, MED30 and the UBE3 paralogs were subjected to additional experimental validation to confirm their regulatory role in MYC expression (Fig. 5).

Upon knockout of the Mediator complex subunit MED30, GFP fluorescence progressively decreased, reaching 78.6% (normalized to NTC) on day 6. In contrast, knockout of UBE3C increased the GFP fluorescence to 168.9% (normalized to NTC, day 6), suggesting an involvement of UBE3C in proteasomal MYC degradation. Given the pronounced positive effect of UBE3C knockout on GFP fluorescence and, hence, MYC expression, we further investigated whether the paralogs of UBE3C (log_2_ β-score (H/I) = 1.0), UBE3A (log_2_ β-score (H/I) = 0.21) and UBE3B (log_2_ β-score (H/I) = 0.10), exerted a similar regulatory function on MYC (Fig. 5C). However, unlike knockout of UBE3C (increased GFP MFI to 225%, normalized to NTC, day 7), knockout of UBE3A and UBE3B had no significant impact on GFP fluorescence, suggesting a specific role of UBE3C in MYC regulation. Given the heterogeneity of multiple myeloma (MM) cells, we tested the effect of the UBE3C knockout on native MYC expression in three different MM cell lines, which were not genetically modified (wild-type (WT), Fig. 5D). Upon UBE3C knockout, in all three WT cell lines MYC expression was noticeably enhanced, confirming that UBE3C is involved in negative MYC regulation.

## Discussion

In this study, we aimed to identify novel regulators of endogenous MYC expression by employing a genome-wide CRISPR/spCas9 loss-of-function screening approach. As a representative model for MYC-driven cancer cells, we used the multiple myeloma (MM) cell line RPMI8226, which had been endogenously modified using our previously established protein tagging method^15^. The major advantage of our approach is the use of an endogenously MYC-tagged GFP reporter cell line enabling physiologically relevant and real-time fluorescence-based monitoring of MYC expression (Fig. 1). To confirm that GFP fluorescence reliably reflects MYC expression, we showed that the endogenous MYC-GFP fusion protein responded appropriately to both pharmacological and genetic manipulation of MYC expression by JQ1 treatment (Fig. 1B) and CRISPR/spCas9-mediated knockout of MYC (Fig. 1D, E), respectively. These findings underscored the suitability of the reporter system for screening. After sorting a genome-wide sgRNA library transduced cell clone into high (H), low (L), and intermediate (I) fractions based on MYC-GFP fluorescence intensity, we used our model to perform a loss-of-function screen to evaluate the effect of particular gene knockouts on MYC expression (Fig. 2). The sgRNAs targeting the knockouts of potential MYC activators genes were enriched in sorted fractions with reduced GFP fluorescence (GFP-low). Among these, the known MYC activator IRF4 and multiple components of the Mediator (MED) complex were prominently represented. Conversely, sgRNAs targeting potential genes acting as MYC repressors were found to be depleted in the GFP-low fraction. Among them FBXW7 was identified in additions to its enrichment in the GFP-high fraction, further supporting its role in MYC suppression (Fig. 2B). As a proof of concept, knockouts of the established MYC regulators IRF4 and FBXW7 were used to validate the tagging and screening approach, resulting in pronounced changes in MYC-GFP fluorescence upon knockout (Fig. 2C, D).

To explore broader functional relationships among identified genes, we performed pathway enrichment analyses (ORA) on potential MYC regulators (activating genes and repressing genes with positive beta scores in low vs. intermediate and high vs. intermediate comparisons, respectively). Notably, potential MYC regulators that were identified by sgRNA depletion (negative beta scores) could not be effectively grouped into clusters due to the relatively low number of identified genes. This may be due to biological effects such as cell cycle arrest or apoptosis triggered by MYC repression, leading to the loss of the majority of respective knockout cells during the screen, particularly after the removal of dead cells before sorting. Given these limitations, and to ensure analytical robustness, we focused our downstream analyses on genes identified through sgRNA enrichment (positive beta scores in both L/I and H/I analysis). This allowed for confident identification of candidate MYC regulators. ORA of these enriched genes identified distinct MYC-activating and -repressing gene clusters, including subunits of the MED complex or genes involved in ubiquitin-dependent, proteasomal degradation (Figs. 3 and 4).

Knockout of MED30, a representative subunit of the Mediator (MED) complex, resulted in a marked reduction of MYC-GFP expression, suggesting that MED30 acts as a positive regulator of MYC transcription. The Mediator complex is a master regulator of transcription that cooperates with general transcription factors and RNA polymerase II (POLR2) to control the initiation and elongation of gene transcription^25^ (Fig. 5A, B). The observed reduction in MYC expression may result from disruption of the Mediator complex upon loss of a single subunit, leading to a broader down-regulation of POLR2-mediated transcription. This assumption is supported by our finding that sgRNAs targeting POLR2 subunits (e.g., POLR2L, log₂ β-score (L/I) = 0.61, data not shown) were enriched in the GFP-low fraction, suggesting a general function of POLR2 in MYC activation (Fig. 2B). However, these data must be interpreted with caution since a general down-regulation of POLR2-transcribed genes is not MYC-specific. Additionally, many different MED subunits were identified by the screen (Fig. 4), indicating that MYC repression generally resulted from a loss of the MED complex integrity. An additional study with more focus on individual MED complex subunits is therefore suggested.

In addition to FBXW7-mediated ubiquitin-ligase activity that is known to destabilize MYC^26–28^, we identified the enrichment of USP17L family members, UBE3C, as well as proteasome subunits and AKIRIN2 as potential MYC repressors; shown by positive beta scores in the GFP-high fraction (Figs. 2B and 4). Considering the short half-life and unstable nature of MYC, it is not surprising that the oncoprotein is extensively controlled by posttranslational processes like ubiquitination and proteasomal degradation^29^. AKIRIN2 was recently described to control the nuclear import of proteasomes to regulate MYC proteins^30^, reinforcing the role of AKIRIN2 in destabilizing MYC by its degradation directly in the nucleus. Our findings also confirm that USP17 family deubiquitinases (DUBs) modulate MYC, as recently shown^31^. However, the human USP17L gene family comprises multiple highly homologous paralogs organized in tandem repeats on chromosome 4p16.1^32^. While most members retain the conserved catalytic residues typical of deubiquitinases, many remain uncharacterized, and functional divergence among paralogs cannot be excluded. Given this high sequence similarity, sgRNAs from pooled libraries such as Brunello may target more than one paralog or the broader USP17L locus, complicating assignment of effects to a single gene. We therefore interpret our results as implicating the USP17L cluster in MYC regulation, with definition of the specific paralog(s) and mechanisms left for future work.

The most remarkable result of our screen was the strong increase in MYC expression upon UBE3C knockout, suggesting its role as a negative MYC regulator. UBE3C belongs to the E3 ubiquitin ligases family that was further described to enhance proteasome processivity by ubiquitinating partially degraded substrates^22^. In MM cell lines, UBE3C was previously shown to be enriched in a tumor-initiating and chemoresistant side population, which is particularly sensitive to proteasome inhibition, highlighting the relevance of the ubiquitin–proteasome system in aggressive MM subpopulations^33,34^. Although the UBE3C function has not previously been linked to MYC expression, its paralog UBE3B was shown to mediate MYC degradation in human lymphoma^24^. In our study, only UBE3C knockout—unlike knockouts of UBE3A or UBE3B—led to increased MYC-GFP levels, indicating a potentially MM-specific role for UBE3C in repressing MYC (Fig. 5B–D). These findings position UBE3C as a novel candidate for future mechanistic and therapeutic studies in MM and possibly other MYC-driven diseases.

In conclusion, we demonstrate the feasibility and reliability of using endogenously tagged MYC reporter cells for functional genomic screening. The identification of known MYC regulators such as FBXW7 and IRF4 affirms the screen’s validity, while the discovery of UBE3C introduces a new candidate for targeted investigation in MYC-driven malignancies. Our observation is consistent with the well-established view that MYC regulation is tightly coupled to global transcriptional and proteostatic control. As a general transcriptional amplifier, MYC depends on core transcriptional cofactors such as the Mediator complex, while its protein stability is governed by the ubiquitin–proteasome system. Accordingly, the identification of these pathways in our screen likely reflects the integration of MYC into fundamental cellular processes, highlighting the central mechanisms that predominantly influence MYC abundance.

Beyond the identification of MYC regulation pathways, our findings provide broader insight into how MYC activity is controlled in MM and how this knowledge might be leveraged therapeutically. MYC integrates multiple signaling and regulatory networks, and our data highlight the importance of both transcriptional co-activators and proteasomal regulators in maintaining MYC homeostasis. Disruption of these regulatory layers may contribute to the persistent MYC activation observed in advanced or therapy-resistant MM. From a therapeutic perspective, indirect targeting of MYC through modulation of its upstream regulators—like UBE3C—could represent a feasible strategy to complement existing MM treatments, particularly those involving proteasome inhibition. Thus, our study expands the understanding of MYC regulation in MM and provides a foundation for exploring new approaches to limit MYC-driven tumor progression.

## Supplementary Information

Supplementary Information 1.

Supplementary Information 2.

Supplementary Information 3.

## Data Availability

The datasets generated and/or analysed during the current study are publicly available. Raw sequencing data have been deposited in the NCBI BioProject repository under accession number PRJNA1354411: https://www.ncbi.nlm.nih.gov/bioproject/?term=PRJNA1354411. Processed datasets are available in the BioGRID ORCS repository (Seibert et al. 2025): https://wiki.thebiogrid.org/doku.php/orcs:prepublication_datasets:seibert2025.
