# Supplementary material for: Endogenous protein tagging coupled with a CRISPR screening approach identifies UBE3C as a potential MYC oncogene regulator
Source: Sci Rep. 2026 Apr 11;16:12194. doi: 10.1038/s41598-026-47974-w (PMC13076874; doi:10.1038/s41598-026-47974-w)
Supplement: Supplementary file 2 — Supplementary Information 2. [file 41598_2026_47974_MOESM2_ESM.docx]

**Supplementary results:**

**Table S1 – Sequences of nLAP tag integrations in selected RPMI8226 GFP-MYC subclones (grey-marked).** In the native MYC allele, the underlined nucleotides symbolize the CRISPR PAM (antisense CCN) sequence directing spCas9-mediated DNA double-strand breaks (DSBs, marked with a slash). The red ATG is the endogenous start codon of MYC; the green ATG is the in-frame start codon of the tagged fusion protein (coming from the hygromycin resistance cassette).

| RPMI8226  clones | PCR A (5’-junction)  5’----------------------------- | PCR B (3’-junction)  ----------------------3’ | PCR C (native MYC)  5’----------------------3’ |
| --- | --- | --- | --- |
| Reference | CCGCGAtccaggGTACCACCatg | cctcCGATGCCCCTC | CCGCGA/CGATGCCCCTC |
| B12 | CCGCGATCCAGGGTACCACCATG | cctcCGATGCCCCTC | CCGCGA/CGATGCCCCTC |
| D4 | CCGCGATCCAGGGTACCACCATG | cctcCGATGCCCCTC | CCGCGA/CGATGCCCCTC |
| F11 | CCGCGATCCAGGGTACCACCATG | cctcCGATGCCCCTC | CCGCGA/CGATGCCCCTC |
|  |  |  |  |


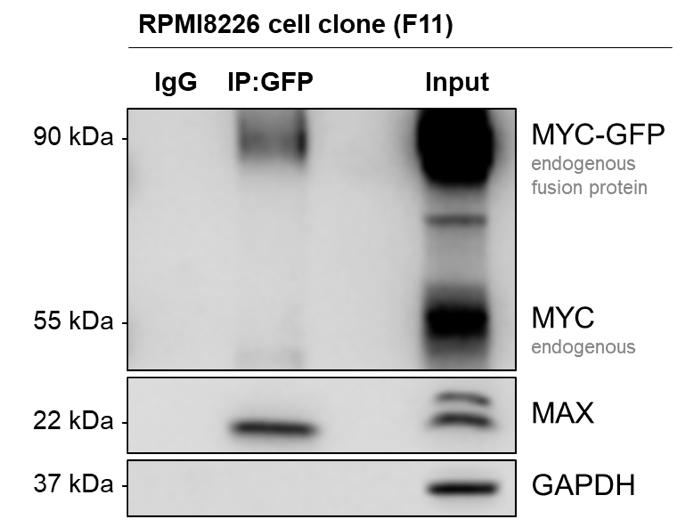


**Figure S1** – **Co-immunoprecipitation (CoIP) of GFP-tagged MYC and its interaction with MAX.** RPMI8226 cell clones (F11) expressing GFP-tagged MYC were lysed, and the lysate was incubated with GFP-Trap® magnetic agarose beads to isolate MYC-interacting proteins. A control immunoprecipitation (IgG) was performed to ensure specificity, showing no background signaling. Input lane represents the protein lysate before precipitation. GAPDH served as control. Proteins were analyzed by SDS-PAGE and Western blotting to detect MYC and MAX interactions.


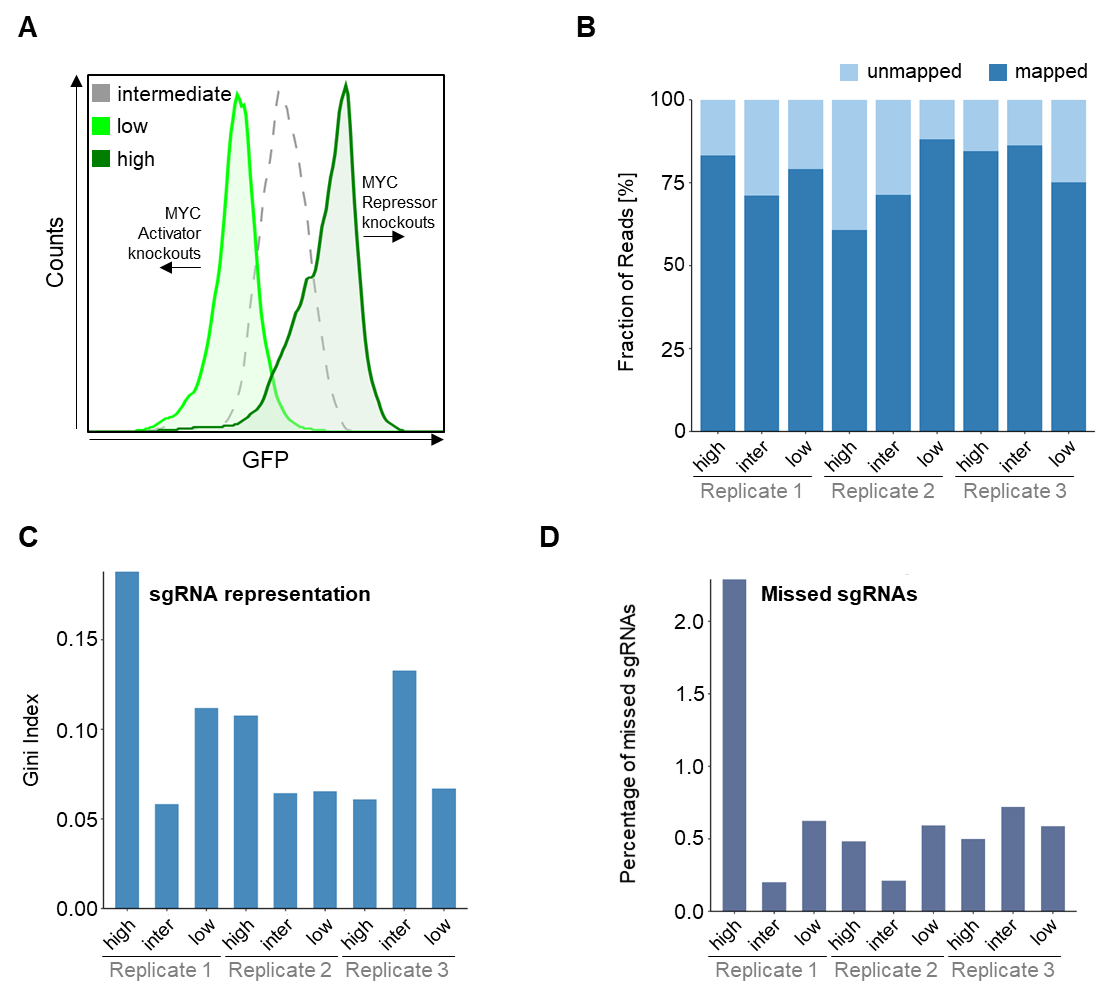


**Figure S2** – **CRISPR screen cell sorting and quality control.** **A** Representative flow cytometry (FACS) profiles of the three sorted fractions from one replicate. The mean fluorescence intensity (MFI) values were 1139 for the intermediate control fraction, 587 for the GFP-low fraction, and 2213 for the GFP-high fraction. Arrows indicate expected changes in GFP fluorescence upon knockout of MYC activators or repressors as shown. Data were analyzed using FlowJo v7.6.5 software. **B** Normalized alignment rates for each sorted fraction across biological replicates. **C** Distribution of sequencing reads across all sgRNAs, represented by the Gini index. **D** Percentage of missing sgRNAs per sample.

**A**

**B**

**Low vs. intermediate**

**High vs. intermediate**

**Figure S3** – **CRISPR screen Volcano plots.** **A** Volcano plots of significantly enriched genes (red marked) in the GFP-low vs. intermediate fraction and **B** of the GFP-high vs. intermediate fraction. Candidate genes that were used for functional gene overrepresentation analysis are marked in pink and red (cut-off value ≥0.36).


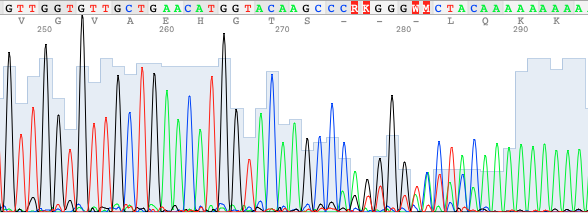

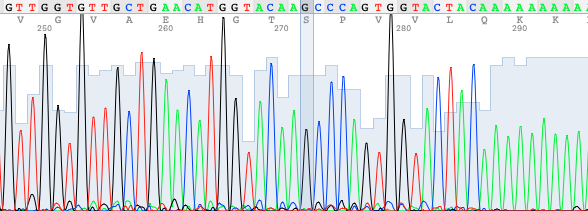


GTTGGTGTTGCTGAACATGGTACAAGCCCAGTGGTActacaaaaaaaaa

**GAACATGGTACAAGCCCAGTGG**

**FBXW7 KO**

**NTC sgRNA**

sgRNA(2):

**FBXW7 WT sequence:**

CRISPR-induced DNA break


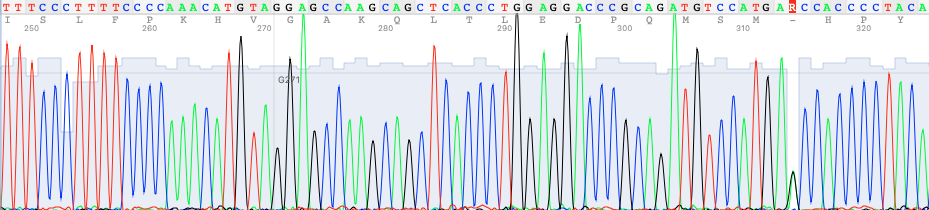

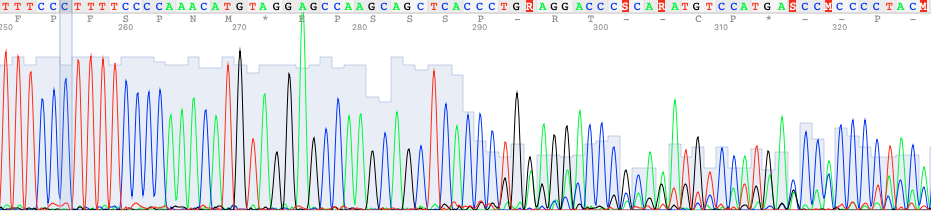


AAACATGTAGGAGCCAAGCAGCTCACCCTGGAGGACCCGCAGATGTCCA

**GCCAAGCAGCTCACCCTGGAGG**

**IRF4 KO**

**NTC sgRNA**

sgRNA(2):

**IRF4 WT sequence**

CRISPR-induced DNA break

**A**

**B**

**C**

**D**


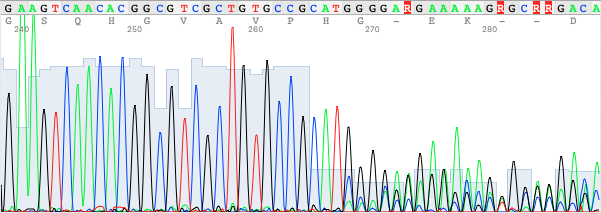

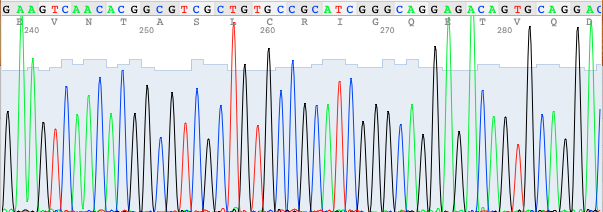


AAGTCAACACGGCGTCGCTGTGCCGCATCGGGCAGGAGACAGTGCAGGA

**CCGCATCGGGCAGGAGACAGTG**

**MED30 KO**

**NTC sgRNA**

sgRNA(2):

**MED30 WT sequence**

CRISPR-induced DNA break

**Figure S4** – **Confirmation of target gene knockout by DNA sequencing and immune blotting. A** CRISPR-induced DNA double strand break (DSB) of FBXW7; **B** DSB of IRF4; and **C** DSB of MED30. For UBE3C, PCR amplification of the sgRNA-targeted genomic region was unsuccessful because highly repetitive sequences adjacent to the cut site prevented reliable amplification and sequencing. **D** Western blot analysis confirming the knockouts of the respective genes. Western blot analysis confirms MYC downregulation in IRF4- and MED30 knockout cells, respectively. Knockout of FBXW7 results in upregulation of MYC. Western blots were probed with an anti-MYC-, anti-FBXW7-, anti-IRF4-, anti-MED30-, and anti-UBE3C antibody; anti-GAPDH served as a loading control.
