# Supplementary material for: Endogenous protein tagging coupled with a CRISPR screening approach identifies UBE3C as a potential MYC oncogene regulator
Source: Sci Rep. 2026 Apr 11;16:12194. doi: 10.1038/s41598-026-47974-w (PMC13076874; doi:10.1038/s41598-026-47974-w)

Uncropped Western Blots

Shown in manuscript

Figure 1 A

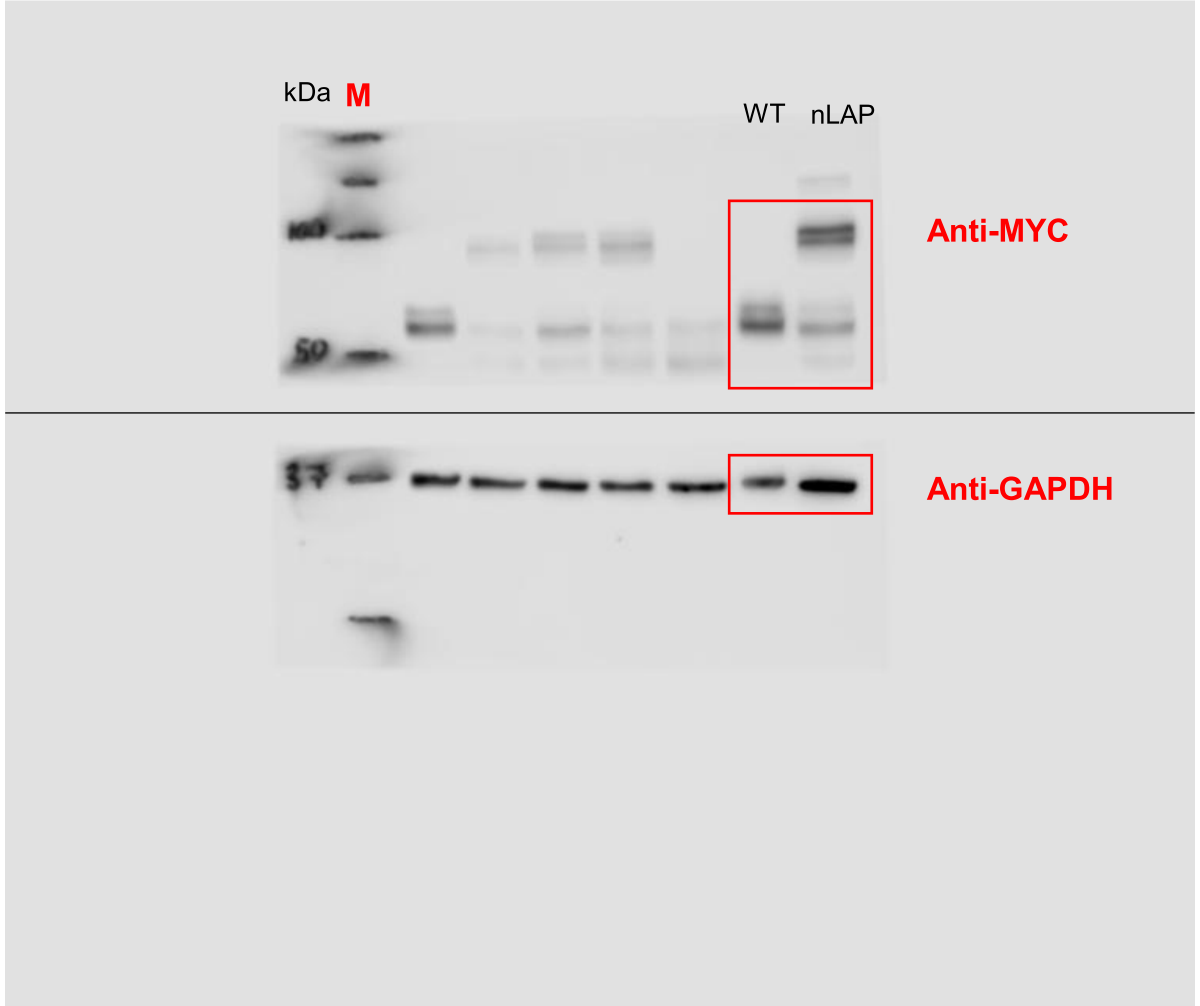

Figure 1 B

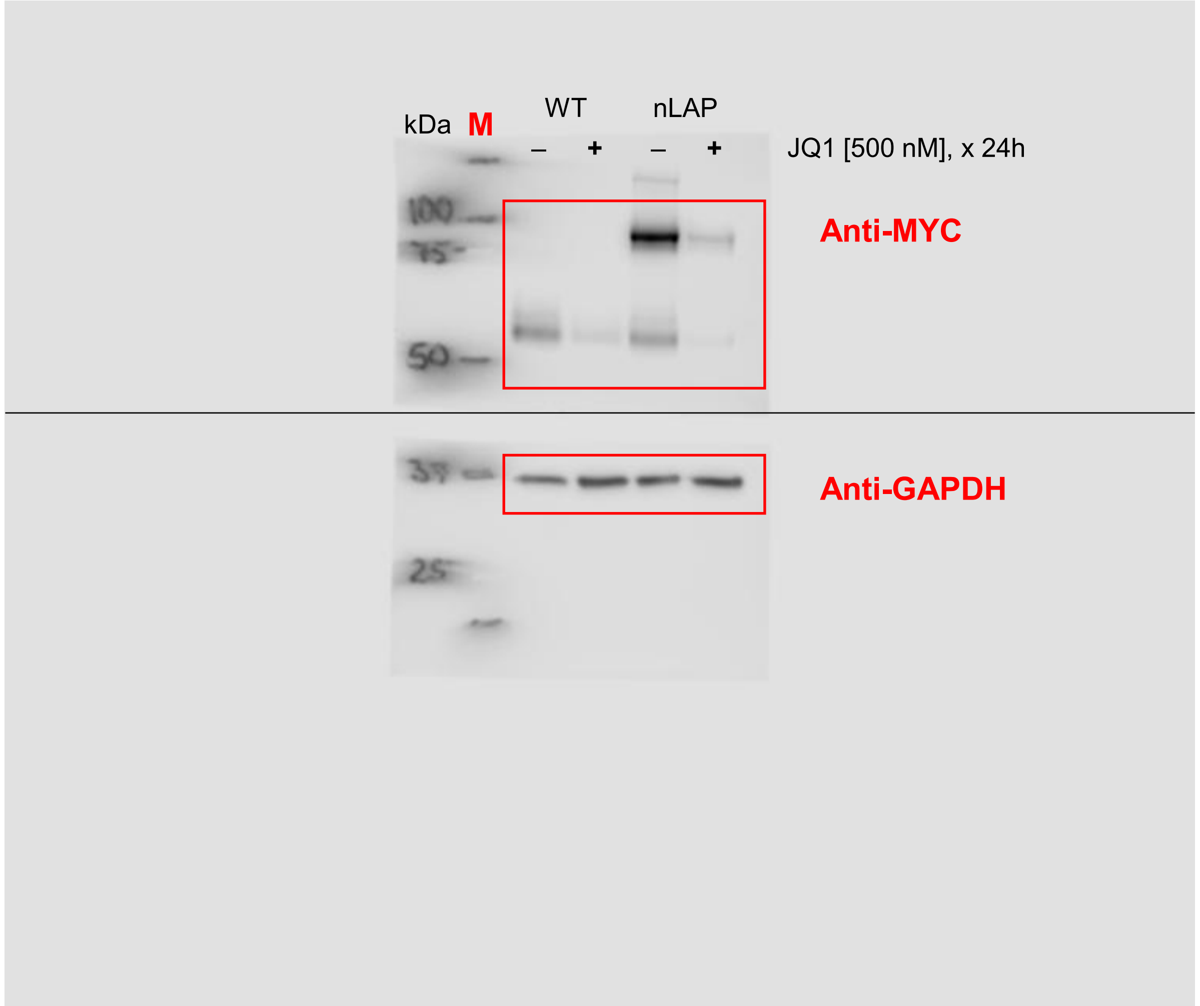

Uncropped Western Blots

Shown in manuscript

Figure 1C

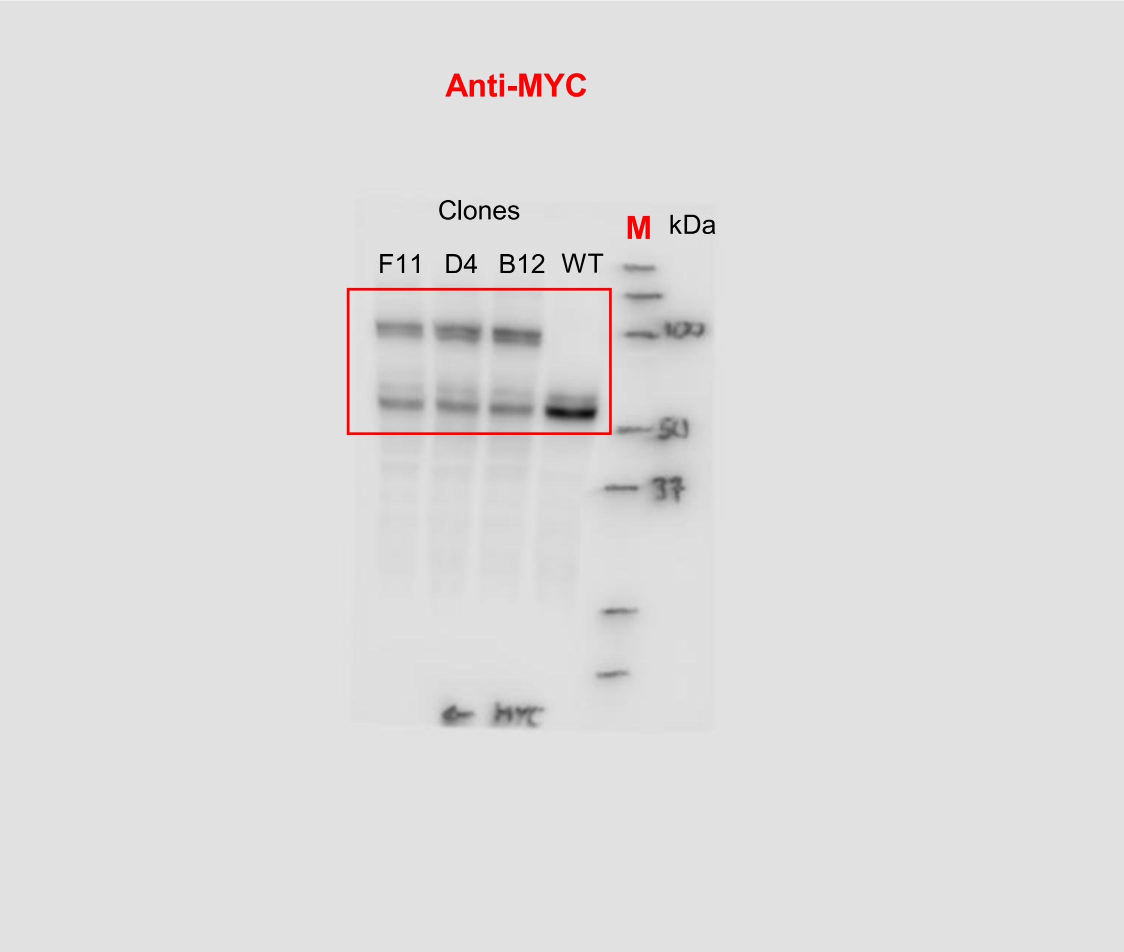

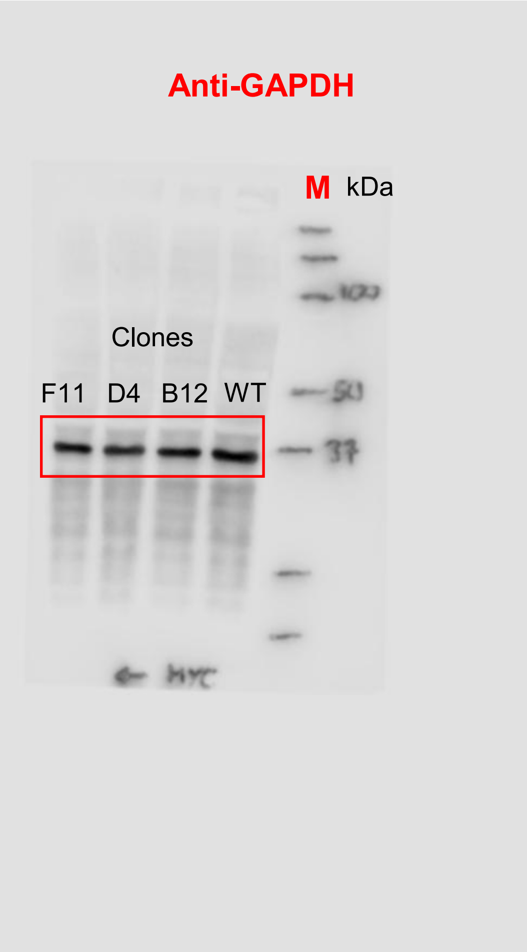

Displayed mirrored

Displayed mirrored

Uncropped Blots

Shown in manuscript

Figure 5D (contrast adjusted)

RPMI8226 WT

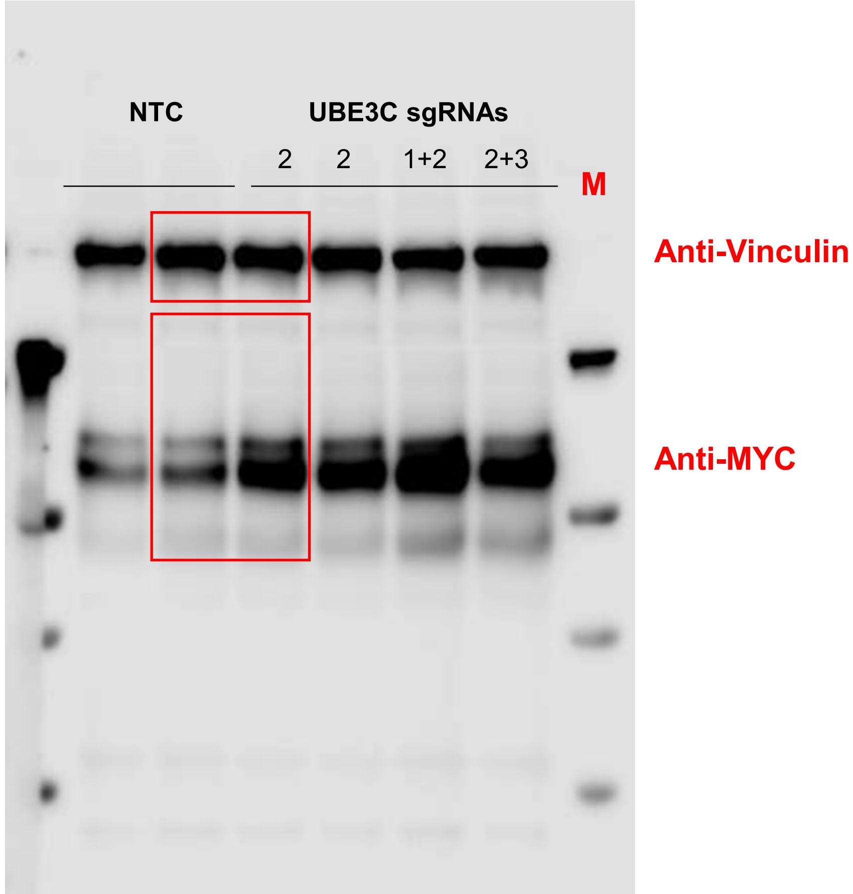

LP1 WT

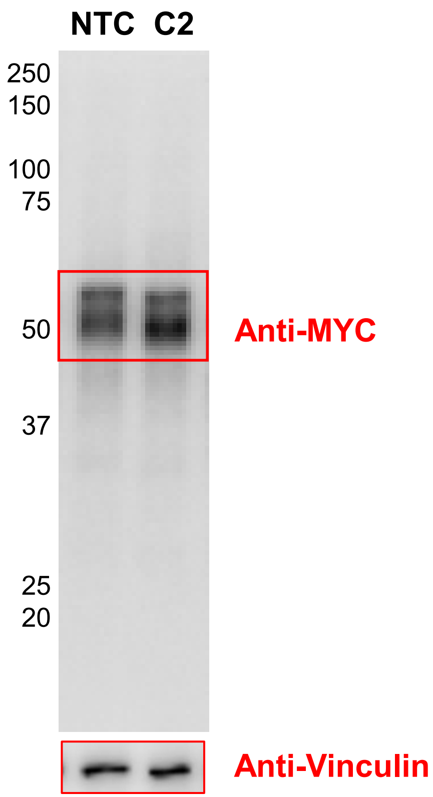

OPM2 WT

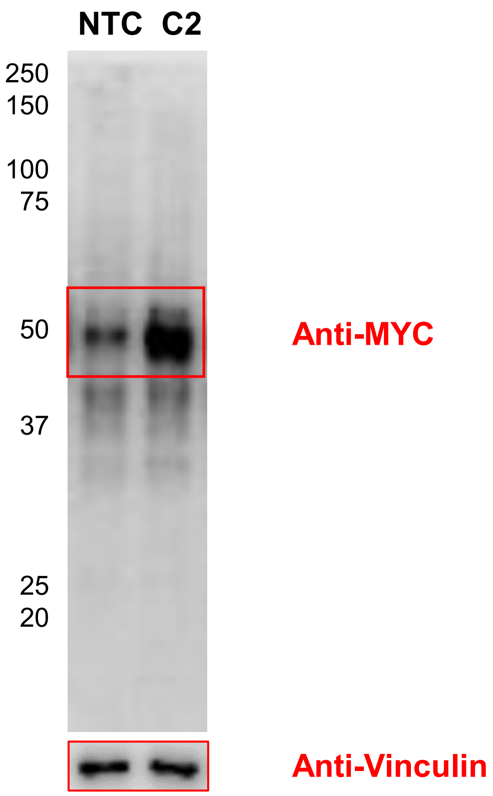

Uncropped Blots

Shown in manuscript

Figure 5D (Lane 4-7)

Lane 1-3 = RPMI8226 (not shown in the manuscript)
Lane 4-5 = LP1
Lane 6-7 = OPM2

NTC/ UBE3C sgRNA(2)

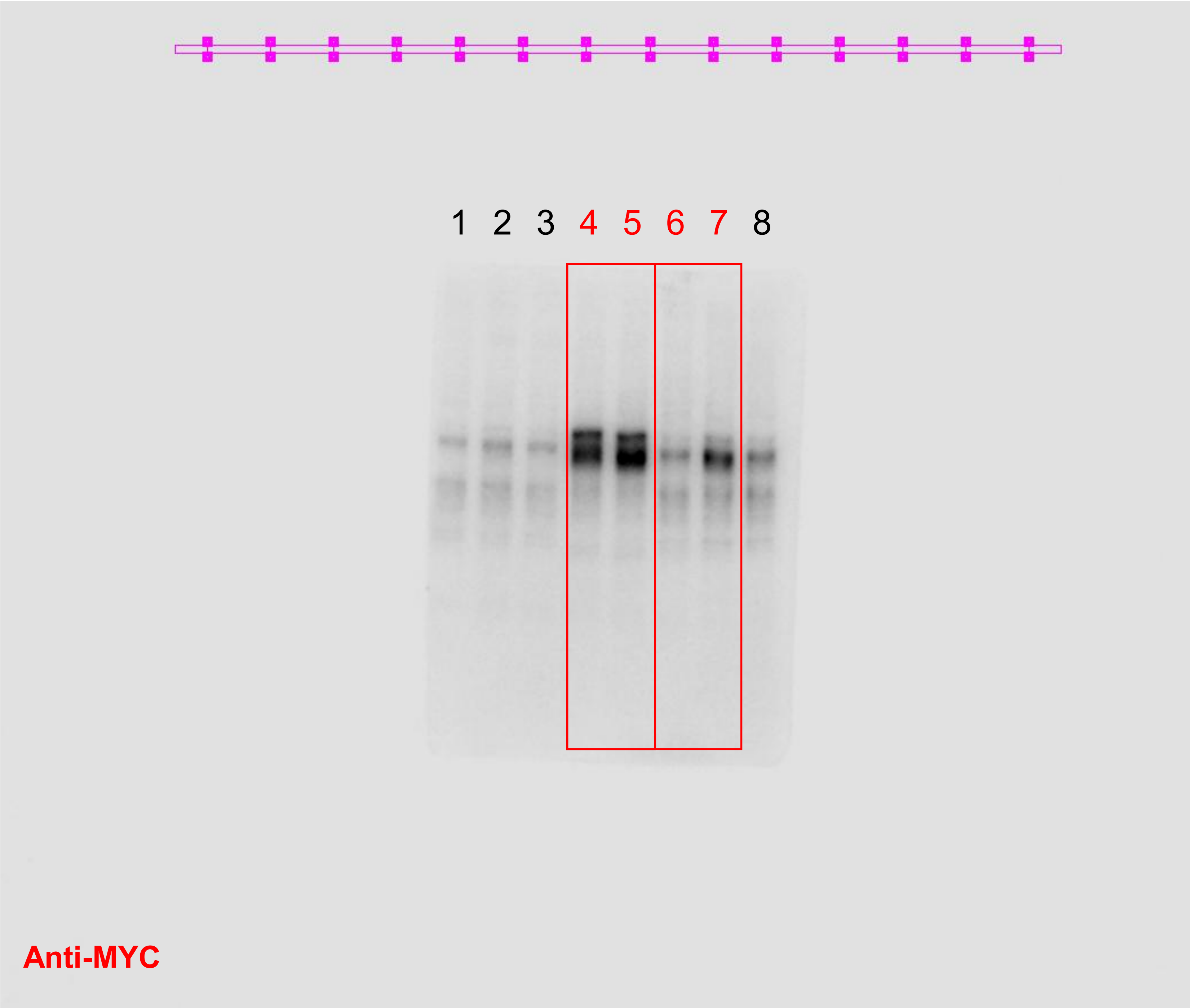

Uncropped Blots

Shown in manuscript

Figure 5D (Lane 4-7)

Lane 1-3 = RPMI8226 (not shown in the manuscript)
Lane 4-5 = LP1
Lane 6-7 = OPM2

NTC/ UBE3C sgRNA(2)

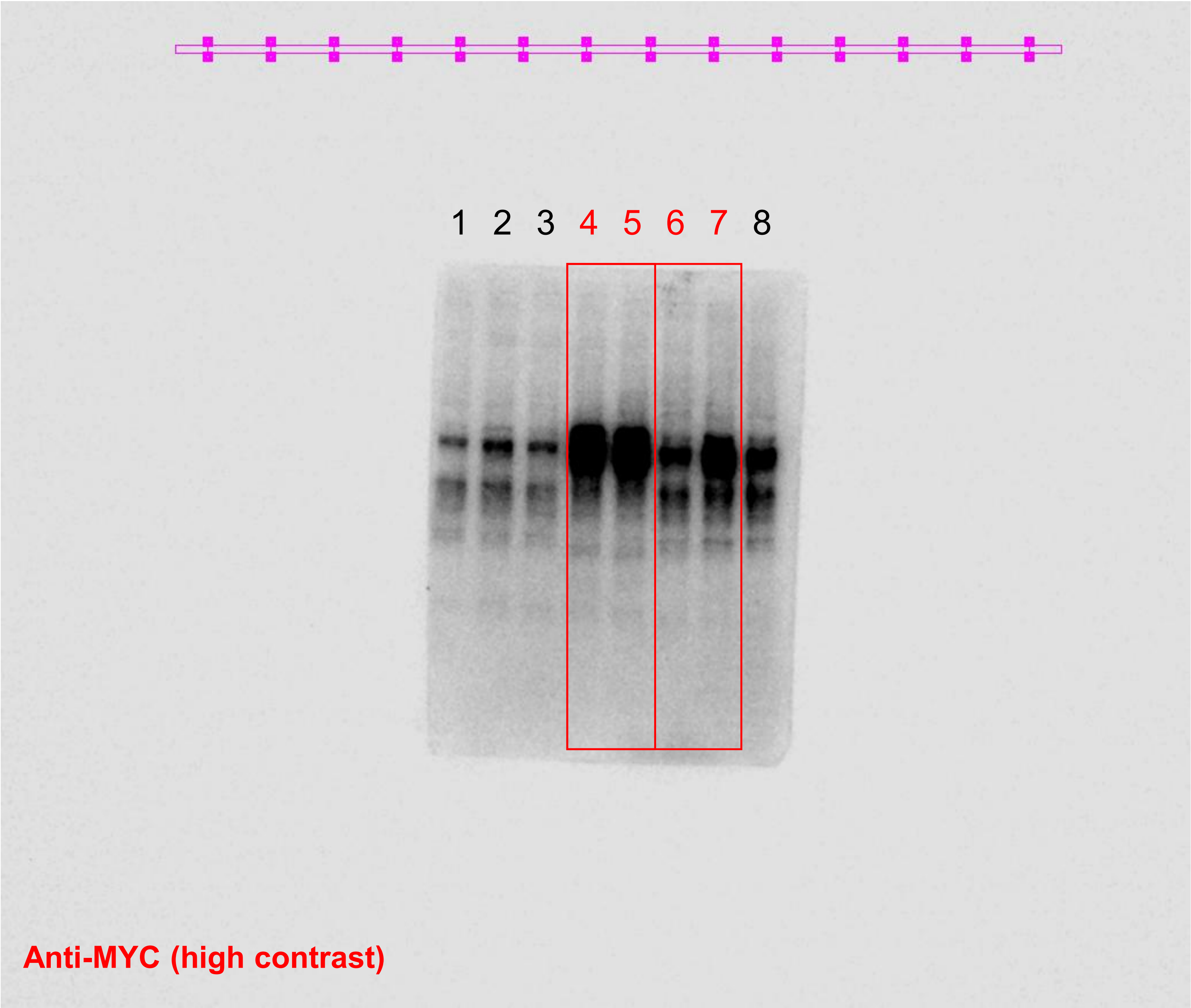

Uncropped Blots

Shown in manuscript

Figure 5D (Lane 4-7)

Lane 1-3 = RPMI8226 (not shown in the manuscript)
Lane 4-5 = LP1
Lane 6-7 = OPM2

NTC/ UBE3C sgRNA(2)

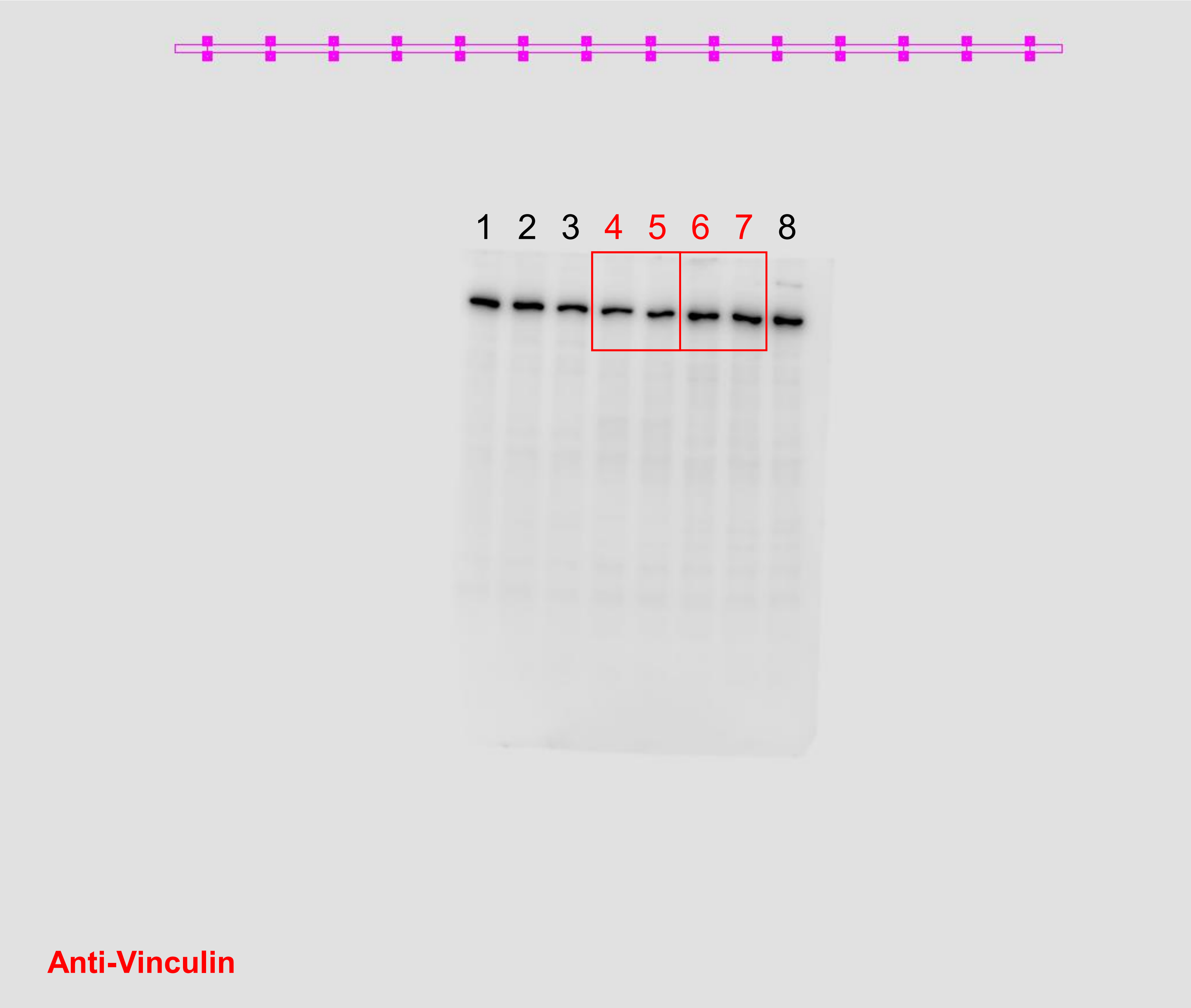

Uncropped Western Blots

Shown in manuscript

Figure S1

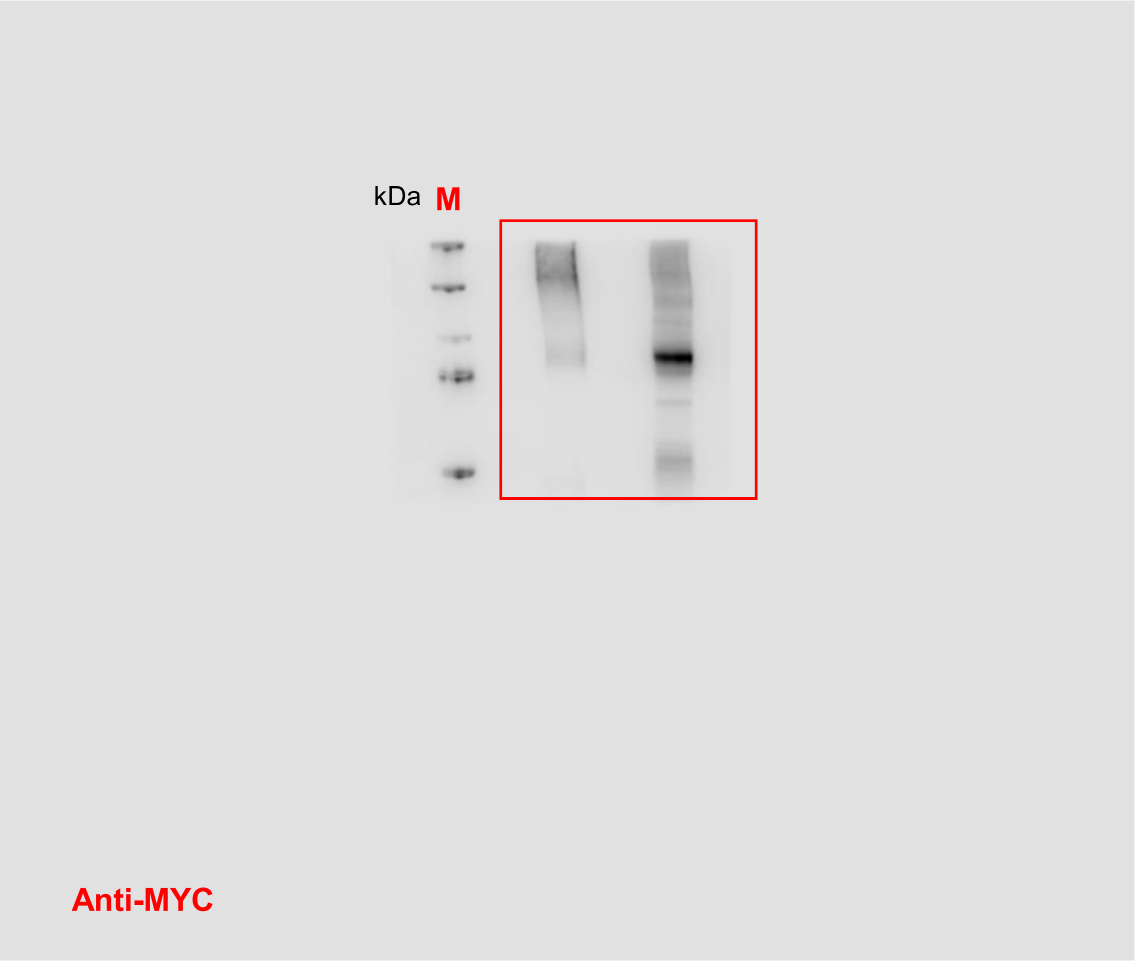

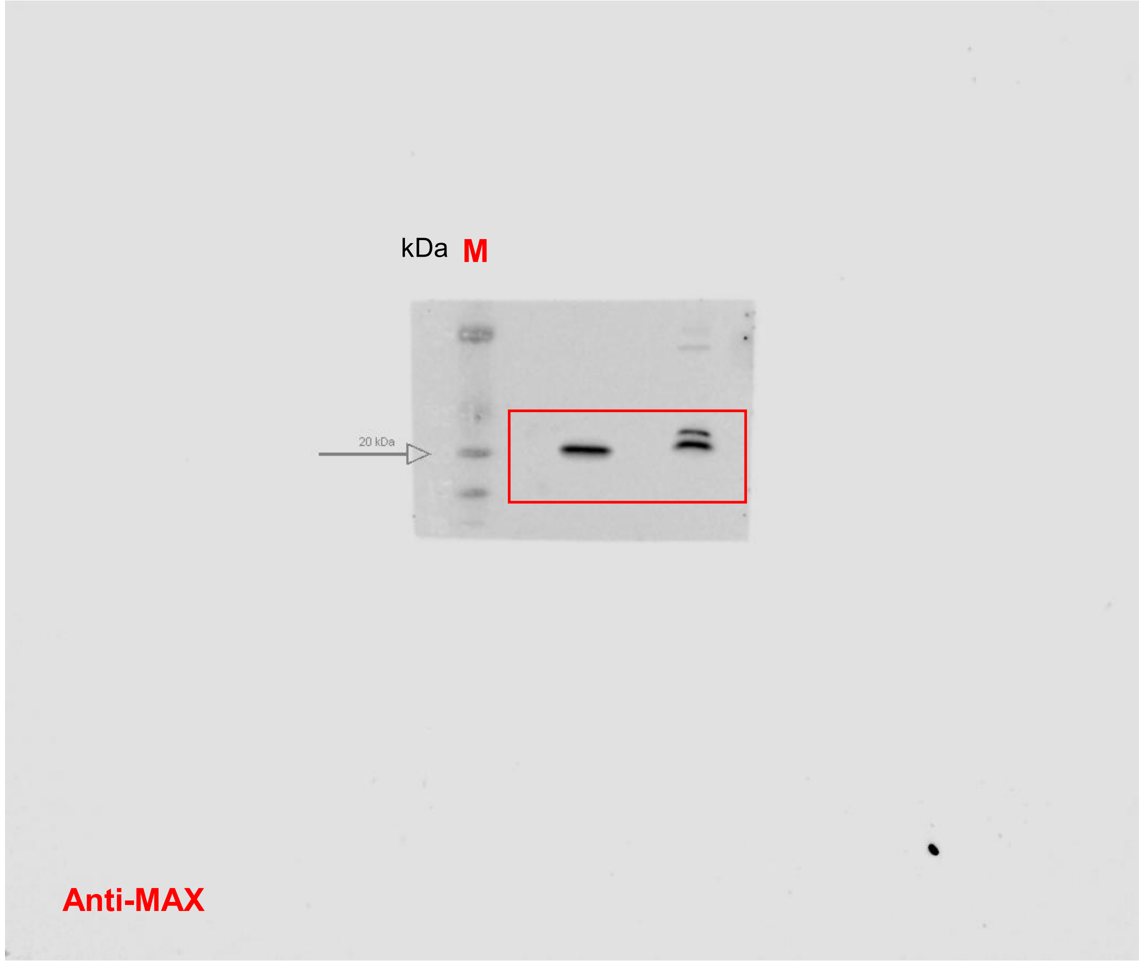

Uncropped Western Blots

Shown in manuscript

Figure S1

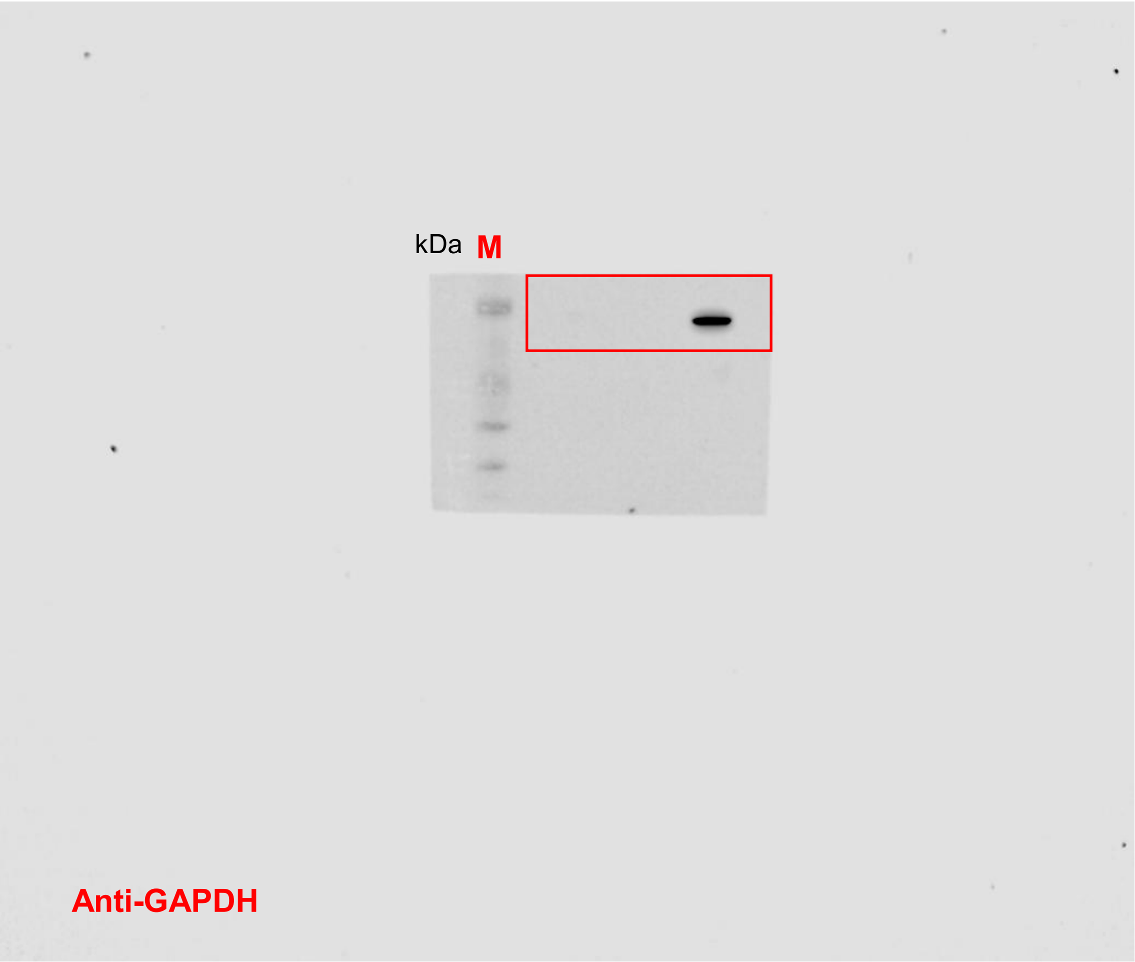

Uncropped Western Blots

Shown in manuscript

Figure S4D

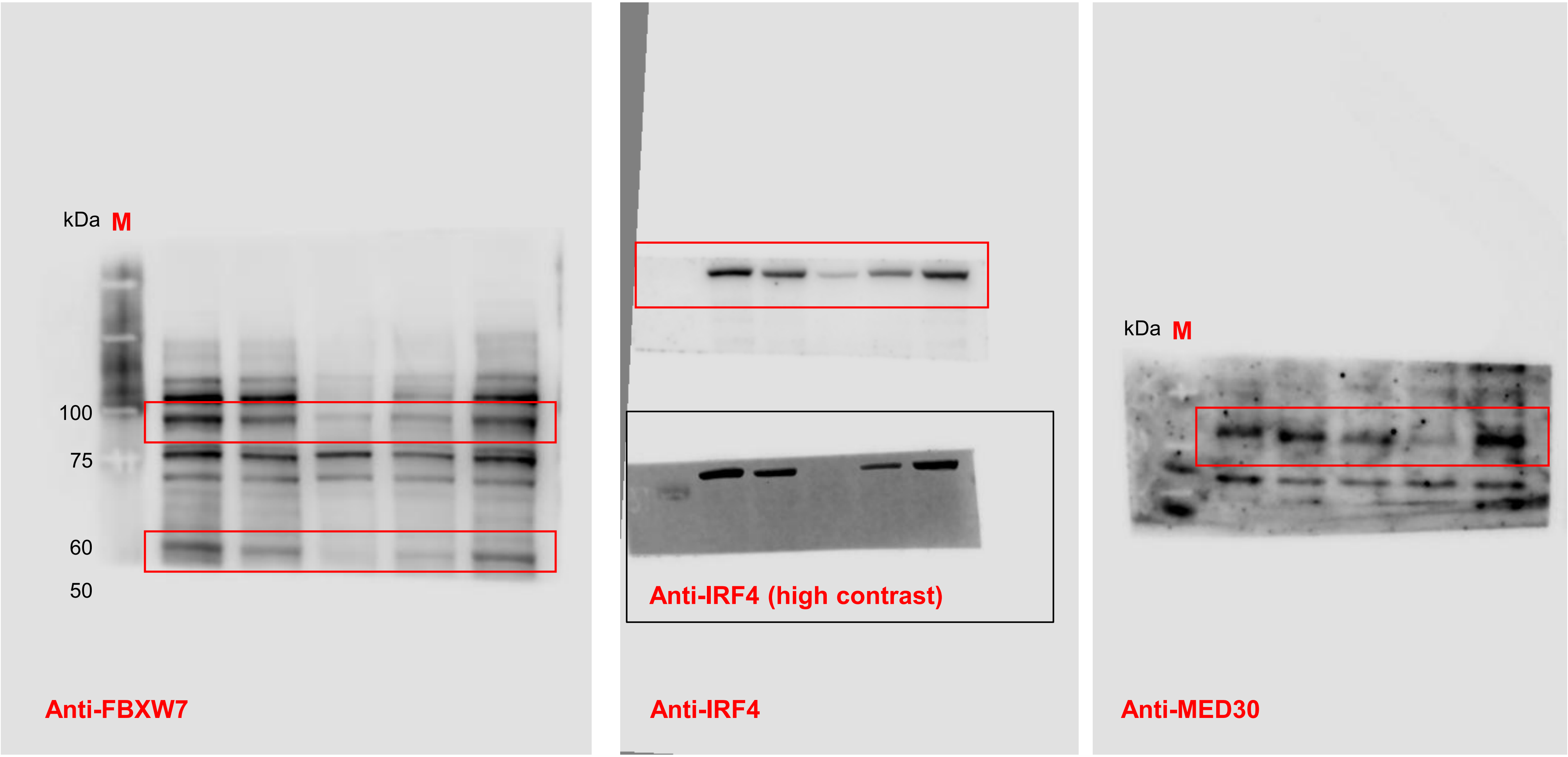

Uncropped Western Blots

Shown in manuscript

Figure S4D

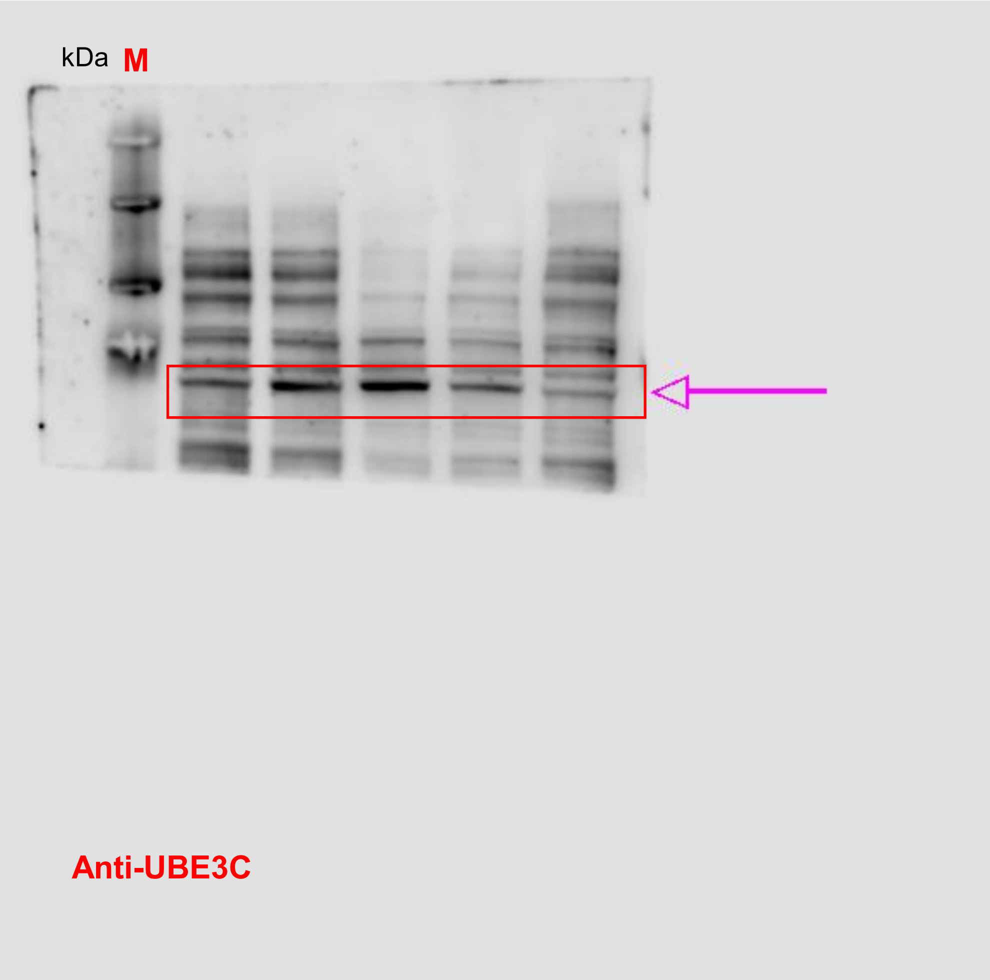

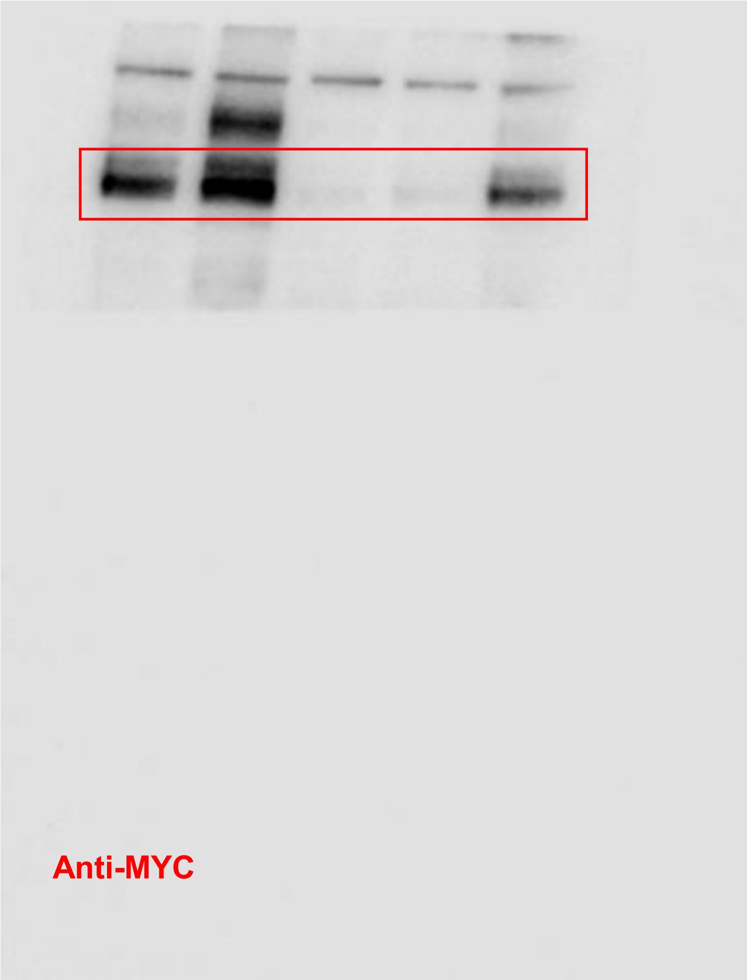

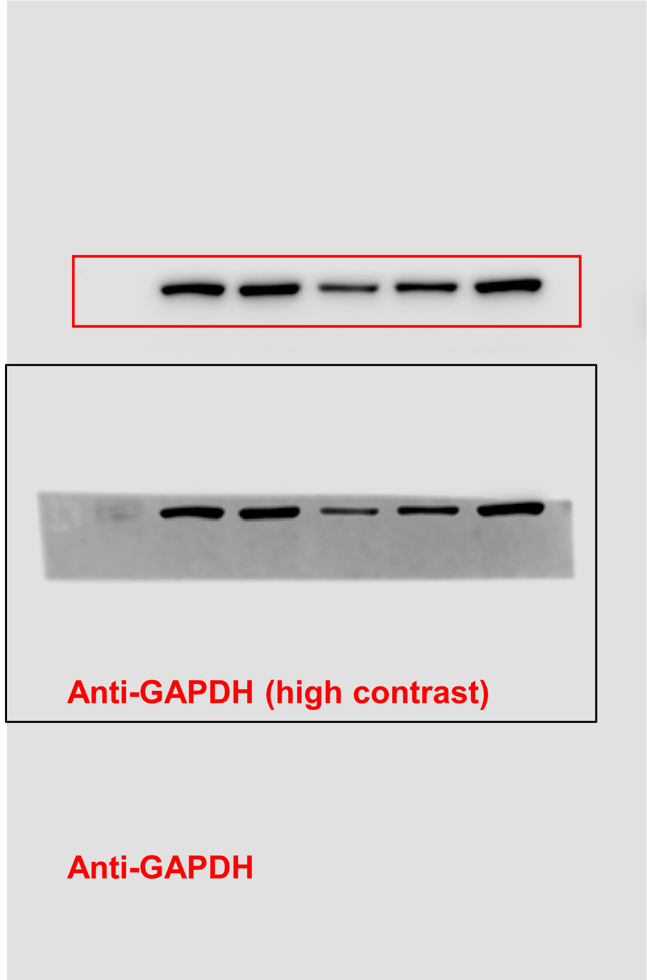

Supplement: Supplementary file 3 — Supplementary Information 3. [file 41598_2026_47974_MOESM3_ESM.pdf]
